# Electron‐Rich, Lewis Acidic Diborane Meets N‐Heterocyclic Aromatics: Formation and Electron Transfer in Cyclophane Boranes

**DOI:** 10.1002/chem.202000189

**Published:** 2020-02-27

**Authors:** Anna Widera, Erik Filbeck, Hubert Wadepohl, Elisabeth Kaifer, Hans‐Jörg Himmel

**Affiliations:** ^1^ Anorganisch-Chemisches Institut Ruprecht-Karls-Universität Heidelberg Im Neuenheimer Feld 270 69121 Heidelberg Germany

**Keywords:** boron, cyclophanes, diborane, redox, viologens

## Abstract

Herein reported are the reactions of an electron‐rich, Lewis acidic diborane with N‐heterocyclic aromatics to give first members of an unprecedented family of highly charged cationic cyclophanes with diboranyl units. Tetracationic cyclophanes with 4,4‘‐bipyridine/ 1,2‐bis(4‐pyridyl)ethylene and diboranyl units were synthesized and their redox chemistry was studied. Cyclisation of two diboranyl and two pyrazine units is accompanied by electron transfer from the diboranyl unit to the pyrazine. Our results pave the way for the integration of redox‐active diboranyl units into cyclophanes and supramolecular structures.

## Graphical abstract

**Electron‐Rich cyclophanes**: Unprecedented, highly charged cyclophanes with diboranyl units are synthesized and their redox and optical properties studied.

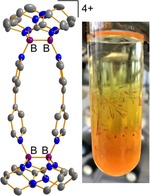

Organic multi‐electron acceptors or donors are desirable and essential materials for a wide range of applications,^1^, ^2^, ^3^ for example, artificial photosystem components, effective redox‐responsive molecular machines or charge storage in batteries. The material class of cyclophanes that incorporate 4,4’‐bipyridinium units (also known as viologens) became valuable candidates for such applications because they not only exist as multi‐cationic species with several accessible mixed‐valence states but also demonstrate self‐assembly properties by incorporating π‐electron‐rich guests.^4^, ^5^, ^6^, ^7^ Figure 1 a shows the Lewis representations of some literature‐known examples of viologen cyclophanes with aromatic or flexible alkyl linkers, cyclobis(paraquat‐*p*‐phenylene) (CBPQT^4+^), cyclobis(paraquat‐*p*‐biphenylene) (CBPQTBP^4+^), and ExBox^4+^, a box‐like cyclophane comprising two 4,4’‐phenylene‐linked „extended“ bipyridinium units.^8^, ^9^, ^10^, ^11^ Very recently, first cyclotris(paraquat‐*p*‐phenylenes) were synthesized.^12^ Cyclophanes with 1,2‐bis(4‐pyridyl)ethylene units, that could be photoisomerized, were also reported.^13^, ^14^ In particular, the redox chemistry and host–guest interactions in these interesting compounds were studied.

**Figure 1:**
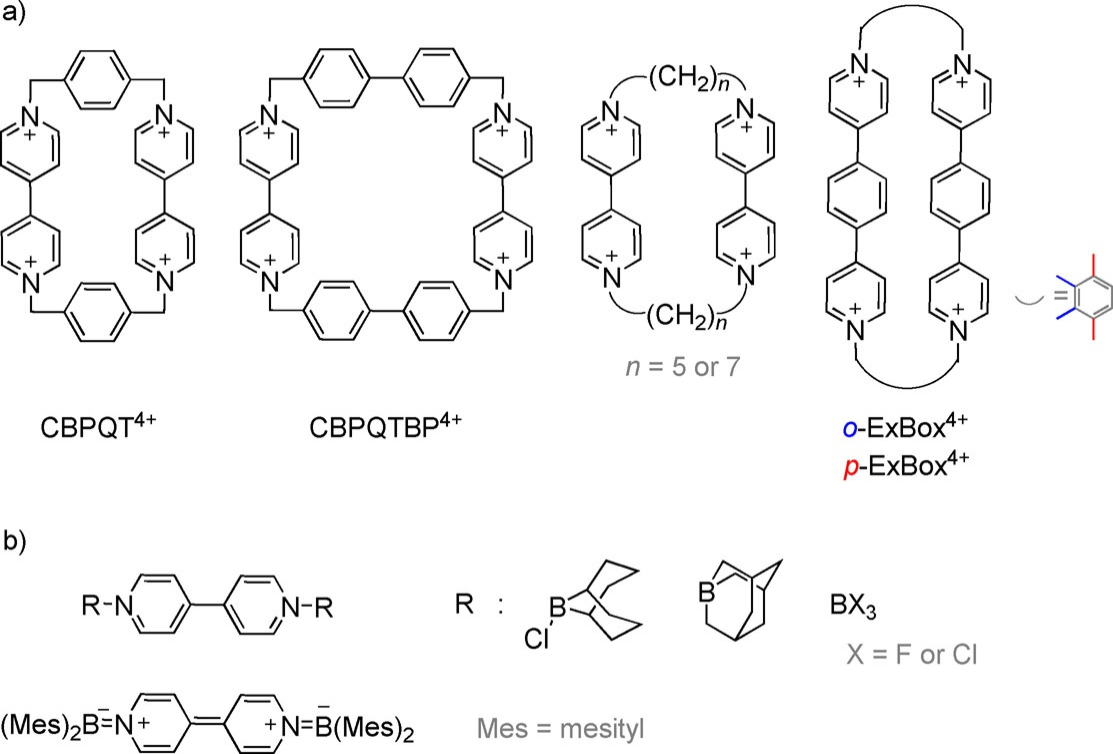
Examples for well investigated cationic bis‐viologen cyclophanes (a) and boron functionalized viologen derivatives (b).

Until now, boron‐containing cyclophanes are very rare, being restricted to compounds with B^III^ atoms,^15^, ^16^, ^17^, ^18^ although first examples for 4,4‘‐bipyridine adducts with simple B^III^ compounds have been reported over 30 years ago (Figure 1 b).^8^ Moreover, a dibora‐compound with the reduced form of this organic linker was synthesized (Figure 1 b).^19^, ^20^ In addition, there are some further examples for complexes of heavier Group 13 homologues (Al, Ga, and In) with bipyridine and pyrazine that exhibit a high structural variety potentially useful for constructing for example, MOFs and other functional materials.^21^, ^22^, ^23^

Motivated by the results and promising properties of cyclophanes, we present herein the first representatives of a new class of easily accessible viologen cyclophanes with cationic redox‐active diborane linkers and investigate on a further macrocycle based on pyrazine. This contribution should extend the repertoire of known boracyclophanes and offer an alternative for possible applications as catalytically redox‐active reaction centers.^24^, ^25^

In our previous investigations we focused on the elimination of the triflate substituents in the diborane **1** and the synthesis of cationic boron species. The abstraction of the triflates in **1** was achieved both with the Lewis acids GaCl_3_ and AlCl_3_ leading to a tetracationic tetraborane^26^, ^27^ and with neutral Lewis bases under formation of diborane dications (Scheme 1).^28^, ^29^

**Scheme 1:**
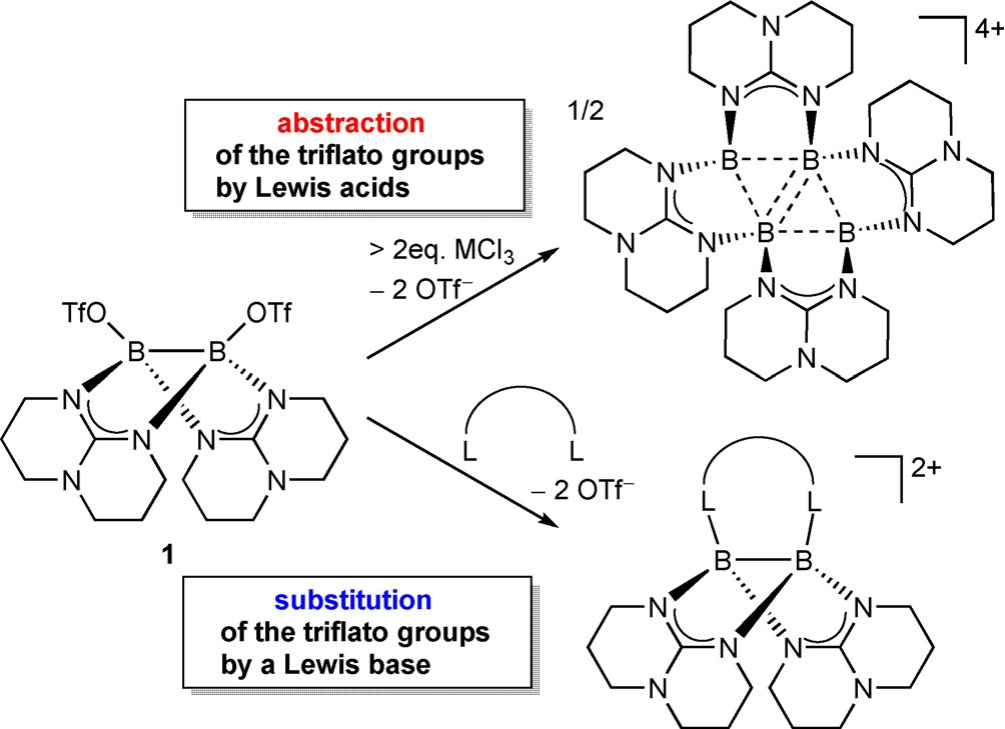
Reaction of the ditriflato diborane **1** with Lewis acids (MCl_3_, M=Al or Ga) and Lewis bases L−L (e.g. 2,2‘‐bipyridine, 1,2‐bis(tetramethylguanidino)‐benzene or phenanthroline).

In previous studies, these abstraction reactions were carried out with Lewis bases that offer two equivalent Lewis basic centers for coordination to the two boron atoms. To elaborate on this issue, we reacted **1** with one equivalent of 1,1‐dimethyl‐1,1‘‐methylene phosphine (dmbp) in CH_2_Cl_2_. This reaction led to the new diboron dication **2** as colorless needles in a yield of 52 % (Scheme 2).

**Scheme 2:**
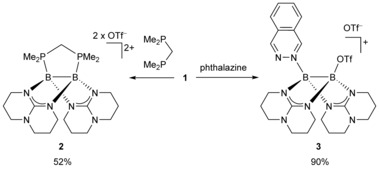
Reactions leading to mono‐ and dicationic diborane(4) compounds.

The recently reported reaction of the highly Lewis acidic tetra(*o*‐tolyl)diborane(4) with phthalazine in toluene yielded the first *N*,*N‘*‐diboryl‐2,3‐dihydrophthalazine (after 10 min reaction time) and then in 8 h 1,2‐bis(borylimidoyl)benzene, in which the N−N bond was completely cleaved.^30^ In contrast, the reaction of **1** with phthalazine resulted in the complexation of one phthalazine molecule to give monocationic diborane(4) derivative **3** in 90 % yield as yellow crystals (Scheme 2). This reaction offers a convenient access to asymmetric diboranes by substitution of the remaining triflato substituent. It should be mentioned that diboron complexes of phthalazine and related compounds are intermediates in boron‐catalyzed (inverse electron demand) Diels–Alder reactions.^31^, ^32^

Two singlets in the ^11^B NMR spectrum of **3**(OTf) at *δ*_exp_=5.9 and 1.9 ppm confirm the asymmetric substitution pattern and are in a good agreement with the quantum chemically calculated values of *δ*_calc_=4.8 and 2.4 ppm (BP86‐D3/def2‐SVP level of theory, see the Supporting Information for spectra). Moreover, the B−B bond length (1.717(6) Å) is similar to that in the starting material **1** (1.708(4) Å),^33^ confirming that no electron transfer to the π‐acidic ligand occurred (Figure 2).

**Figure 2:**
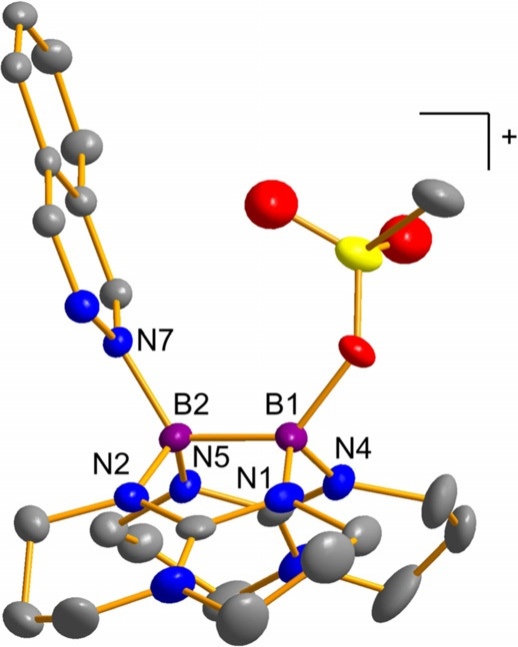
Experimental structure of the monocationic diborane **3** obtained by reaction of **1** with phthalazine. Only one set of the disordered OTf moieties is shown. All hydrogen and fluorine atoms are omitted for clarity. Displacement ellipsoids are drawn at the 50 % probability level. Selected bond lengths in Å: B1−B2 1.717(6), B1−N1 1.528(7), B1−N4 1.521(6), B2−N2 1.529(6), B2−N5 1.529(6), B2−N7 1.591(6).

The three N‐heterocyclic aromatics 4,4‘‐bipyridine (bpy), 1,2‐di(4‐pyridyl)ethylene (dpe) and pyrazine (pz) were in the focus of the following experiments. Reacting compound **1** with bpy or dpe resulted in the formation of an unprecedented tetracationic tetraboron cyclophane (Scheme 3 a). During the reactions an interesting color change of the mixture was observed. Directly after addition of the solvent, the solution adopted a dark‐green color. Solution EPR spectra recorded at this stage showed the presence of organic radicals (Figure S21, left in the Supporting Information). The obvious inference is that electron transfer from the electron‐rich diborane to the organic electron acceptor generated radical anions. After some time, the reaction again changed its color (to orange‐red), the EPR signal vanished and the final products were formed. To investigate on this phenomenon, we changed the solvent to the less polar THF. A dark violet and extremely air sensitive solid precipitated immediately after addition of bpy indicating the possible formation of a one‐electron‐reduced viologen exhibiting a characteristic blue‐violet color.^34^ The EPR spectrum of the solid at room temperature (Figure S21, right) proves the paramagnetic character of the intermediate compound with a value of *g=*2.003.

**Scheme 3:**
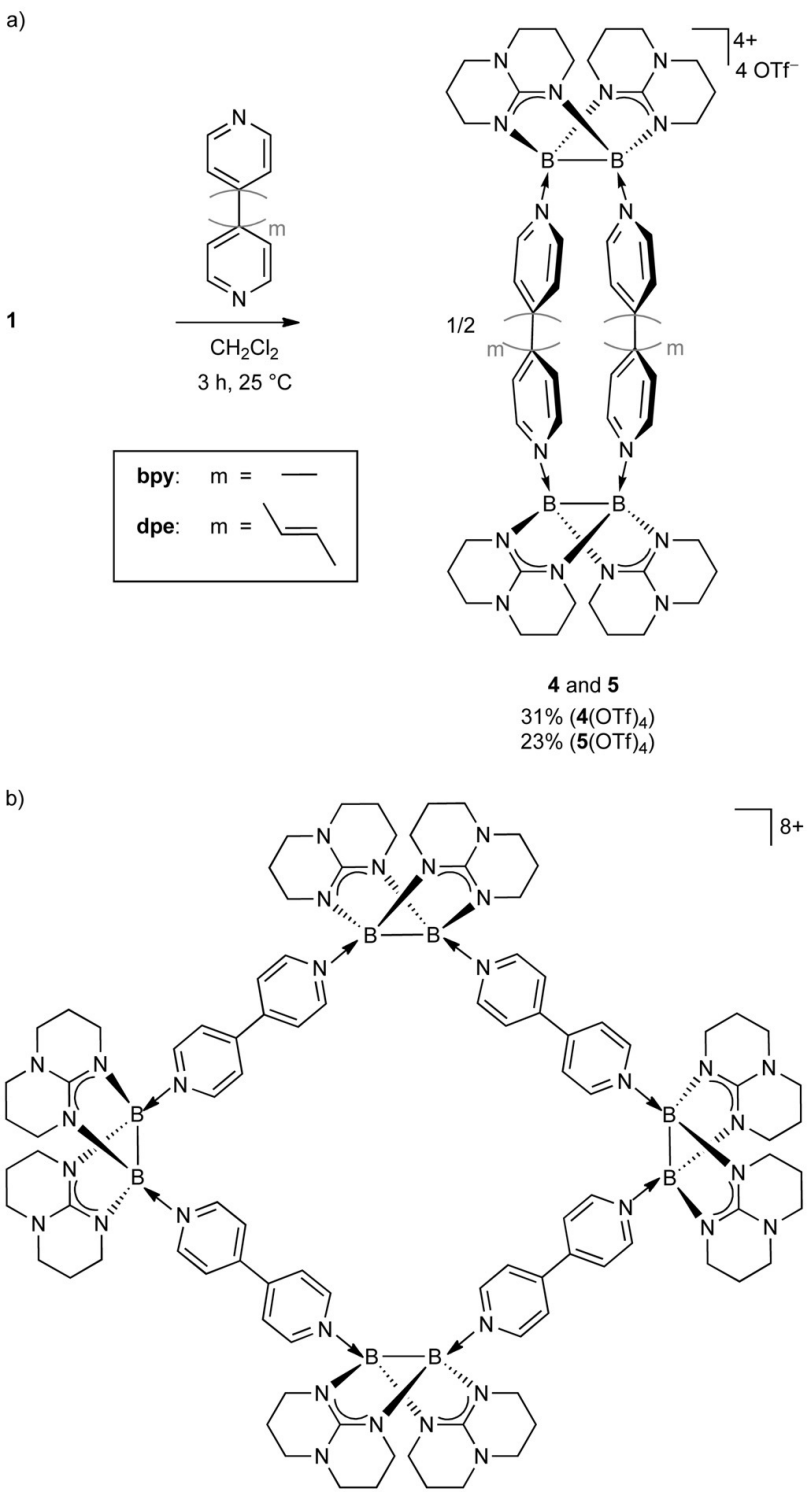
a) Synthesis of the first bis‐viologen cyclophane with two diborane linkers (**4** and **5**) and b) Lewis representation of the crystallized octacationic byproduct in the reaction with bpy.

Both compounds, **4**(OTf)_4_ and **5**(OTf)_4_, were crystallized from a 1:1 mixture of methylene chloride and *n*‐pentane giving deep‐red needles (Figure 3) in 31 and 23 % yield, respectively. Crystals of **4**(OTf)_4_ grew likewise from a concentrated aqueous solution demonstrating the quite remarkable stability of the tetracation towards hydrolysis. Due to the persistent disorder of the triflate anions it was not possible to obtain satisfying crystal structures both of **4**(OTf)_4_ and **5**(OTf)_4_. To overcome this difficulty, we replaced the anion in a metathesis reaction with four equivalents of Na[B(C_6_F_5_)_4_]. The experimentally determined structure of the tetracations **4** and **5** are shown in Figure 3.

**Figure 3:**
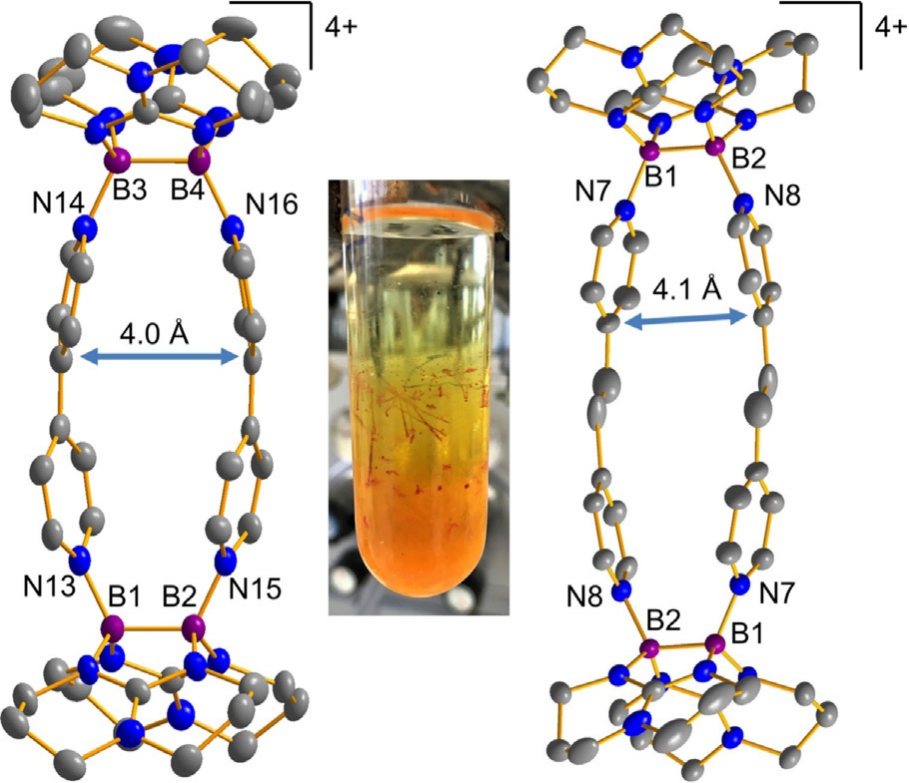
Left: Illustration of the experimentally determined structure of the cyclophane tetracation **4** formed in the reaction of **1** with 4,4‘‐bipyridine followed by metathesis with Na[B(C_6_F_5_)_4_]. Only one set of the disordered atoms is shown. Displacement ellipsoids are drawn at the 50 % probability level. Selected bond lengths in Å: B1−B2 1.728(8), B3−B4 1.724(8), B1−N13 1.587(7), B2−N15 1.575(7), B3−N14 1.594(7), B4−N16 1.603(7). Middle: Photo of the crystalline **4**(OTf)_4_. Right: Illustration of the experimentally determined structure of **5** formed in the reaction of **1** with 1,2‐di(pyridyl)ethylene followed by metathesis with Na[B(C_6_F_5_)_4_]. Displacement ellipsoids are drawn at the 20 % probability level. Selected bond lengths in Å: B1−B2 1.7244(0), B1−N7 1.5665(0), B2−N8 1.5998(0). All hydrogen atoms and the four [B(C_6_F_5_)_4_]^−^ anions are omitted for clarity.

The interaction of two viologen cations or dications was previously studied theoretically.^35^ The experimentally determined solid‐state centroid‐to‐centroid distance between the two dicationic 4,4‘‐bipyridyl units in **4** amounts to only 4.0 Å, in line with the quantum chemically calculated value of 3.9 Å (B3LYP‐D3/def2‐TZVPP). The observed distance is significantly shorter than the value of 6.8 Å previously determined for the well‐known CBPQT^4+^ (Figure 1) and comparable to the 3.5 Å measured for the related *o*‐ExBox^4+^ system.^36^, ^37^

Further NMR studies clearly showed that both compounds **4**(OTf)_4_ and **5**(OTf)_4_ are stable in solution. The ^11^B NMR spectra in [D_3_]acetonitrile display in both cases one singlet signal and the chemical shifts are very similar (*δ*=3.9 ppm for **4** and *δ*=3.2 ppm for **5**).

The experimental UV/Vis spectrum of **4**(OTf)_4_ (Figure S20 in the Supporting Information) displays one broad absorption band in the visible region (*λ*_max_=397 nm) and an additional sharp band at 251 nm. A comparison with the carbon analogue, methylviologen (MV^2+^) points to an intriguing similarity. The parent dication MV^2+^ possesses only one strong absorption band at 257 nm, close to the value measured for **4**.^38^, ^39^ After two‐electron reduction, one band at 395 nm was measured.^40^ Given that the reported wavelength and the value recorded for **4** are closely conform we postulate a boron‐to‐ligand charge transfer.

We investigated on the nature of the interactions between the linkers by cyclic voltammetry (Figure 4, top). Given that the cyclophane **4** contains two bpy units, up to four one‐electron reduction steps can occur.^11^ In the cyclic voltammogram of a CH_2_Cl_2_ solution three waves were observed, that were assigned to two reversible one‐electron reduction processes (at *E*_1/2_=−1.10 and −1.30 V) followed by a reversible two‐electron reduction process at *E*_1/2_=−1.71 V, converting the initial tetracation eventually to a neutral molecule.

**Figure 4:**
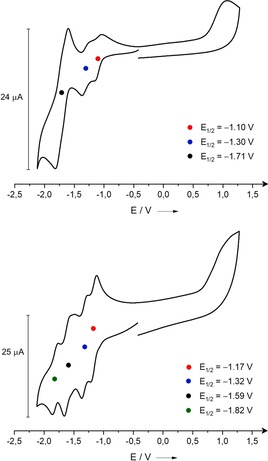
The cyclic voltammograms of **4**(OTf)_4_ (top) and **5**(OTf)_4_ (bottom). A glassy carbon working electrode, a platinum counter electrode and an Ag/AgCl reference electrode were used to characterize 4.23×10^−1^ m (**4**(OTf)_4_) and 5.84×10^−1^ m (**5**(OTf)_4_) solutions in CH_2_Cl_2_ at 298 K with 0.1 m *n*Bu_4_NPF_6_ as electrolyte. Scan rate of 100 mV s^−1^ was used for this analysis and the obtained values were referenced to the ferrocenium/ferrocene (Fc^+^/Fc) redox couple.

We observed a further wave splitting of the reversible two‐electron couple in CH_3_CN solution exhibiting a reduction peak separation of 230 mV, presumably a consequence of the steric and electronic constraints associated with the rigid geometry of the cyclophane (Figure S22 in the Supporting Information).^11^ In the cyclic voltammogram of **5**, the last two‐electron reduction process is clearly separated in two one‐electron steps (Figure 4, bottom). The relatively large potential differences between the redox events argue for significant electronic coupling between the two viologen units. In the case of *o*‐ExBox^4+^, the two reduction steps (one‐electron redox processes) are separated by 516 mV.^37^ The analogue with two viologen linkers, CBPQT^4+^, shows no interaction between the aromatic systems as no wave splitting occurs in the CV and only two two‐electron processes are visible.^41^ Based on these observations we rank the strength of the electronic interactions in **4** and **5** between those in *o*‐ExBox and CBPQT.

Motivated by the promising cyclic voltammograms we attempted a chemical reduction of the tetracation **4** by reacting it with an excess of zinc in CH_3_CN. During the reaction we observed a slight color change to orange brown, but only isolated the starting material. We also tried to isolate the side products involved in the formation of **4**(OTf)_4_ by crystallization from the concentrated mother liquor. Indeed, a few yellow box‐shaped crystals were obtained at room temperature. The X‐ray analysis revealed a tetrameric octacationic macrocycle as byproduct (Scheme 3 b) being formally the dimerization product of **4**. Unfortunately, we could not obtain a satisfying crystal structure, again due to the disorder of the eight triflate counterions. Nevertheless, the quality of the data for the cationic part was quite gratifying (Figure S23 in the Supporting Information) demonstrating that even larger macrocyclic structures are possible. The solution NMR spectra of the reaction mixture showed no clear evidence for an equilibrium between **4** and the tetrameric species at room temperature, but small amounts of the dimer of **4** were observed together with five triflate counterions in an high‐resolution ESI^+^ mass spectrum recorded after heating of **4**(OTf)_4_ in CH_2_Cl_2_. Traces of the trimeric hexacation and the pentamer decacation were detected as well (see Figure S24 for spectra).

Next, we reacted **1** with one equivalent of pyrazine. The product **6**(OTf)_2_, isolated in a yield of 17 %, turned out to be again a tetraboron ring (Scheme 4). However, this time we isolated a dicationic complex in which two oxidized diborane units are linked by two reduced pyrazine ligands (Scheme 4 and Figure 5). The latter exhibits a non‐planar, „boat‐like“ structure caused by the loss of aromaticity upon reduction and possibly also strain.

**Scheme 4:**
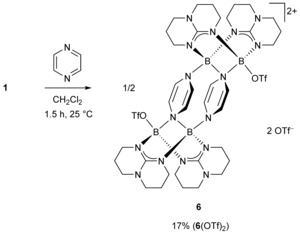
Reaction between the ditriflato‐diborane **1** and pyrazine, leading to a ring structure with reduced pyrazine substituents (**6**).

**Figure 5:**
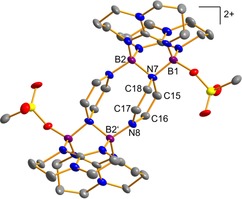
Illustration of the experimentally determined structure of the dication **6**. Displacement ellipsoids are drawn at the 30 % probability level. Only one set of the disordered hpp and OTf moieties is shown. Hydrogen and fluorine atoms are omitted for clarity. Selected bond lengths in Å: B1−N7 1.548(4), B2−N7 1.601(5), B2‘−N8 1.525(4), C15−C16 1.340(5), C17−C18 1.334(5), B1⋅⋅⋅B2 2.453(5).

The electron transfer occurs from the B−B bond (B^II^→B^III^) which at last is cleaved. Consequently, the boron atoms of each diborane unit are now separated by 2.45 Å. In this context it should be noted that complexation coupled with electron transfer was previously observed in some reactions of the ditriflato‐diborane **1**.^28^ Given that pyrazine and 4,4’‐bipyridine have similar redox potentials, these results are in line with our observations for the reaction with 4,4‘‐bipyridine.^42^

Compound **6**(OTf)_2_ decomposes in polar solvents like acetonitrile, DMF, or DMSO. Among commonly used organic solvents, it is only stable in dry, degassed methylene chloride. Unfortunately, the solubility of the compound is very low. Therefore, it was only characterized by solid‐state analytics and UV/Vis spectroscopy. In the UV/Vis spectrum, a broad band at 449 nm appeared, indicating reduction of the 1,4‐diazine and agreeing with the solid‐state structure. The value recorded for **6**(OTf)_2_ is 92 nm redshifted in comparison to the absorption band reported for 1,4‐bis(dimesitylboryl)‐1,4‐dihydropyrazine, *λ*=357 nm).^20^ Given that the distance between the reduced pyrazine linkers is only 2.84 Å, there might as well be a significant interaction between the π‐electrons of the organic ligand units.

Following our systematic approach, we reacted the bistriflate **1** with a mixed organic system containing both the pyrazine and bipyridine units, 2,3‐di‐2‐pyridinyl‐pyrazine (dpp). As we observed in the past, the diborane dication forms Lewis acid–base adducts with 2,2‐bipyridine whereas pyrazine becomes reduced.^28^ The combined reactivities in dpp were investigated concerning the formation of a tetraborane diradical species when two equivalents of **1** are reacted with one equivalent of the organic ligand (Scheme 5). However, the product turned out to be the salt **7**(OTf)_2_ (formed in ca. 70 % yield), featuring only one diborane unit. The B−B bond (1.7131(3) Å) was confirmed by structural analysis (Figure 6).

**Scheme 5:**
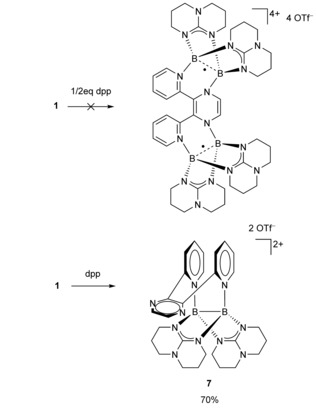
Reaction of **1** with 2,3‐di‐2‐pyridinyl‐pyrazine (dpp) to the dicationic diborane(4) **7**. The formation of a tetracationic compound with two (partially oxidized) diboranyl units was not observed.

**Figure 6:**
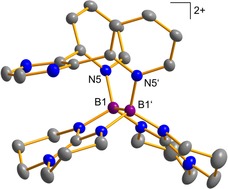
Illustration of the structure of the dication **7**. Displacement ellipsoids are drawn at the 30 % probability level. Only one set of the disordered hpp moieties is shown. Hydrogen atoms are omitted for clarity. Selected bond lengths in Å: B1−B1’ 1.7135(3), B1−N5 1.5942(4).

In summary we presented here the synthesis of new remarkably stable, highly charged tetraboron ring compounds being the first cyclophanes with integrated diborane units. Given that the spatial separation of the organic linkers is small due to the constraints imposed by the diboryl units, there is a significant degree of electronic communication. In the case of pyrazine, ring formation is accompanied by electron transfer, leading to pyrazine reduction and oxidation of the boron atoms (B^II^→B^III^). The methodology reported herein might be used for the synthesis of a variety of new redox‐active cyclophanes.

## Conflict of interest

The authors declare no conflict of interest.

## Supporting information

As a service to our authors and readers, this journal provides supporting information supplied by the authors. Such materials are peer reviewed and may be re‐organized for online delivery, but are not copy‐edited or typeset. Technical support issues arising from supporting information (other than missing files) should be addressed to the authors.

Supplementary
