# Supplementary material for: Electron‐Rich, Lewis Acidic Diborane Meets N‐Heterocyclic Aromatics: Formation and Electron Transfer in Cyclophane Boranes
Source: Chemistry. 2020 Feb 27;26(15):3435–40. doi: 10.1002/chem.202000189 (PMC7155121; doi:10.1002/chem.202000189)
Supplement: Supplementary file 1 — Supplementary [file CHEM-26-3435-s001.pdf]

CHEMISTRY
A European Journal

Supporting Information

Electron-Rich, Lewis Acidic Diborane Meets N-Heterocyclic Aromatics: Formation and Electron Transfer in Cyclophane Boranes

Anna Widera, Erik Filbeck, Hubert Wadepohl, Elisabeth Kaifer, and Hans-Jörg Himmel*[a]

chem_202000189_sm_miscellaneous_information.pdf

Table of Contents

General details on the synthetic work

Experimental Procedures

Analytical Data

Details of the quantum-chemical calculations

General details on the synthetic work

All reactions are carried out both under a dry Ar atmosphere by using standard Schlenk technique and in a glove box (MBraun LABmaster dp, MB-20-G) under dry N_2 atmosphere.
1,1-Dimethyl-1,1`-methylene phosphine (dmbp), phthalazine (phaz), 4,4´-bipyridine (bpy), 1,4-diazine (daz), 2,3-di-2-pyridinyl-pyrazine (dpp) and 1,2-di(4-pyridyl)ethylene (dpe) are purchased from Abcr and Sigma Aldrich and used without further purification. $[TfOB(hpp)]_2$ (**1**)[1] is synthesized according to the literature. All solvents are rigorously dried by applying standard procedures and stored over molecular sieves (4 or 3 Å) after being degassed by the freeze-pump-thaw method. Infrared spectra are recorded as KBr disks with a BIORAD *Excalibur FTS 3000*. BRUKER *Avance II 400*, BRUKER *Avance III 500* and BRUKER *Avance III 600* devices are used for NMR spectroscopy. NMR spectra are recorded at 298 K, if not stated otherwise, and ^{11}B chemical shifts are given relative to $BF_3 \cdot Et_2O$. Elemental analysis is performed at the Microanalytical Laboratory of the University of Heidelberg using the *vario EL* and *vario MICRO cube* devices from Elementar Analysensysteme GmbH. HR-ESI$^+$ mass spectra are obtained with JEOL *JMS-700 magnetic sector* and BRUKER *ApexQe hybrid 9.4 T FT-ICR* at the MS-Laboratory of the University of Heidelberg. The EPR spectra (X band, 9.5 GHz) are recorded in solution and at room temperature with the BRUKER *ESP 300 E* device. *Varian Cary 5000 spectrometer* was used for UV-Vis spectroscopy. The samples were measured in a 1 cm thick quartz cuvette.
Suitable crystals for single-crystal structure determination were taken directly from the mother liquor, taken up in perfluorinated polyether oil and fixed on a cryo loop. Intensity data were collected at low temperature (100 K and 120 K) with the diffractometers *Kappa CCD* (Mo-K_α radiation, compound **7**$(OTf)_2$) and BRUKER *D8 Venture* (Cu-K_α and Mo-K_α radiations, microfocus X-ray tubes, PHOTON III detector, compound **5**$[B(C_6F_5)_4]_4$ and the octacationic octaborane byproduct).
Full shells of intensity data for compounds **3**(OTf), **4**$[B(C_6F_5)_4]_4$ and **6**$(OTf)_2$ were collected at low temperature with an *Agilent Technologies Supernova-E CCD* diffractometer (Cu-K_a radiation, microfocus X-ray tube, multilayer mirror optics). Detector frames (typically ω-, occasionally φ-scans, scan width 1°) were integrated by profile fitting.[2,3] Data were corrected for air and detector absorption, Lorentz and polarization effects[3] and scaled essentially by application of appropriate spherical harmonic functions.[3–5] Absorption by the crystal was treated numerically (Gaussian grid).[5,6] An illumination correction was performed as part of the numerical absorption correction.[5]
The structures were solved by “modern” direct methods[7] with dual-space recycling (compound **4**$[B(C_6F_5)_4]_4 \cdot 4\ CH_2Cl_2$),[8] or by the charge flip procedure (compounds **3**(OTf) and **6**$(OTf)_2$)[9] and refined by full-matrix least squares methods based on F^2 against all unique reflections.[10] All non-hydrogen atoms were given anisotropic displacement parameters. Hydrogen atoms were input at calculated positions and refined with a riding model.[11]
Split atom models were used to refine disordered groups and/or solvent molecules. When found necessary, suitable geometry and adp restraints or constraints were applied.[11,12]
Electron density attributed to solvent of crystallization (methylene chloride) was removed from the structure of **4**$[B(C_6F_5)_4]_4 \cdot 4\ CH_2Cl_2$ with the BYPASS procedure,[13] as implemented in PLATON (squeeze/hybrid).[14] Partial structure factors from the solvent masks were included in the refinement as separate contributions to F_{calc}.
CCDC 1959615 - 1959619 contains the supplementary crystallographic data for this paper. These data can be obtained free of charge from the Cambridge Crystallographic Data Centre's and FIZ Karlsruhe’s joint Access Service via https://www.ccdc.cam.ac.uk/structures/?.

Experimental Procedures

$\{[B(\mu\text{-hpp})]_2(dmbp)\}(OTf)_2$ (**2**$(OTf)_2$): Diborane ditriflate $[TfOB(\mu\text{-hpp})]_2$ (**1**) (40 mg, 0.067 mmol) was dissolved in 1.5 mL of absolute degassed methylene chloride. 1,1-Dimethyl-1,1`-methylene phosphine (dmbp) (10 mg, 0.074 mmol, 12 µL) was added to the solution and the reaction mixture was stirred for 7 hours at room temperature upon formation of a colorless precipitate. The solvent and unreacted phosphine were removed in vacuo and the residue was redissolved in 2.0 mL of methylene chloride. Colorless crystals of the product (27 mg, 0.035 mmol, 52% yield) were obtained by overlaying the methylene chloride solution with 5.0 mL of *n*-pentane. C,H,N analysis (%) for $C_{21}H_{38}N_6B_2P_2O_6S_2F_6 \cdot 0.5$ DCM (774.73 g mol^{-1}): calcd. C 33.33, H 5.07, N 10.85; found C 33.48, H 4.78, N 10.66. $^1H\{^{11}B\}$ NMR (400 MHz, CD_2Cl_2): δ = 3.38–3.17 (m, 16H, N-CH_2), 3.02 (t, 2H, CH_2), 2.08–1.90 (m, 8H, CH_2), 1.71–1.68 (m, 12H, CH_3) ppm. ^{11}B NMR (128 MHz, CD_2Cl_2): δ = 0.25 (s, 2B) ppm. $^{11}B\{^1H\}$ NMR (128 MHz, CD_2Cl_2): δ = 0.25 (s, 2B) ppm. ^{13}C NMR

(101 MHz, CD_2Cl_2): *δ* = 159.5 (C_q, hpp), 121.4 ($SO_3CF_3^-$), 47.1 (NCH_2), 44.4 (NCH_2), 29.9 (PCH_2), 22.2 (CH_2), 8.9 (CH_3) ppm. ^{19}F NMR (376 MHz, CD_2Cl_2): *δ* = –78.88 ($SO_3CF_3^-$) ppm. MS (HR-ESI$^+$): m/z (%) = 583.2335 ({[B(μ-hpp)]$_2$(dmbp)}(OTf)$^+$, 100%). ^{31}P NMR (162 MHz, CD_2Cl_2): *δ* = 1.62 (br, 2P) ppm. IR (KBr): $\tilde{\nu}$ = 3053 (vw), 2953 (m, C_{hpp}-H), 2920 (w, C_{hpp}-H), 2876 (m, C_{hpp}-H), 1602 (s), 1540 (s), 1474 (w), 1398 (m), 1374 (m), 1324 (s), 1259 (s), 1224 (s), 1151 (s), 1049 (s), 1030 (vs), 969 (s), 958 (s), 917 (s), 850 (s), 755 (s), 731 (s), 689 (m), 636 (vs), 572 (s), 517 (vs), 483 (w), 460 (w) cm^{-1}.

{[B(μ-hpp)]$_2$(phaz)(OTf)}(OTf) (**3**(OTf)): Diborane ditriflate [TfOB(μ-hpp)]$_2$ (**1**) (22 mg, 0.037 mmol) and phthalazine (phaz) (5 mg, 0.039 mmol) were put in a dry nitrogen flushed Schlenk flask and dissolved in 0.6 mL of absolute degassed methylene chloride. The reaction mixture immediately turned yellow and was further stirred for 2 hours at room temperature. The solvent was removed in vacuo and the product dried in vacuo to give an isolated yield of 90% (24 mg, 0.033 mmol). Yellow crystals suitable for an X-ray analysis formed by overlaying the methylene chloride solution of **3**(OTf) with *n*-pentane. The compound decomposes in acetonitrile. C,H,N analysis (%) for $C_{24}H_{30}N_8B_2S_2O_6F_6$ (726.30 g mol^{-1}): calcd. C 39.69, H 4.16, N 15.43; found C 39.65, H 4.89, N 14.95. $^1H\{^{11}B\}$ NMR (400 MHz, CD_2Cl_2): *δ* = 9.61 (s, 1H, NCH_{arom}), 9.45 (s, 1H, NCH_{arom}), 8.36 (d, $^3J_{HH}$ = 7.9 Hz, 1H, CH_{arom}), 8.22 (m, 2H, CH_{arom}), 8.20–8.15 (m, 1H, CH_{arom}), 3.46–3.16 (m, 16H, N-CH_2), 2.20–1.93 (m, 8H, CH_2) ppm. ^{11}B NMR (128 MHz, CH_2Cl_2): *δ* = 5.90 (s, *B*OTf), 1.89 (s, *B*N) ppm. $^{11}B\{^1H\}$ NMR (128 MHz, CH_2Cl_2): *δ* = 5.90 (s, *B*OTf), 1.89 (s, *B*N) ppm. ^1J CH-Korr (HSQC) NMR (400 MHz, CD_2Cl_2): *δ* = 153.4 (C_{arom}-H), 149.3 (C_{arom}-H), 137.0 (C_{arom}-H), 135.0 (C_{arom}-H), 128.8 (C_{arom}-H), 127.1 (C_{arom}-H), 47.0 (NCH_2), 39.8 (NCH_2), 38.9 (NCH_2), 21.6 (CH_2), 21.3 (CH_2) ppm. ^{19}F NMR (376 MHz, CH_2Cl_2): *δ* = –79.20 (BSO_3CF_3), –78.85 ($SO_3CF_3^-$) ppm. MS (HR-ESI$^+$): m/z (%) = 577.2304 (**3**, 57%). IR (KBr): $\tilde{\nu}$ = 3322 (w), 3247 (w), 3174 (w), 3052 (w), 2962 (w, C_{hpp}-H), 2877 (w, C_{hpp}-H), 1633 (s), 1604 (s), 1551 (s), 1448 (m), 1381 (m), 1325 (s), 1276 (s), 1155 (s), 1097 (m), 1062 (m), 1030 (s), 915 (w), 868 (w), 800 (m), 758 (m), 637 (vs), 573 (m), 517 (s), 473 (w) cm^{-1}. Crystal data for **3** · 2 DCM, M_r = 896.15, 0.28 x 0.06 x 0.03 mm, triclinic, space group P–1 (*IT* Nr. 2), *a* = 8.64632(19), *b* = 14.1403(3), *c* = 16.6248(2) Å, *α* = 102.6445(15), *β* = 95.7961(15)°, *γ* = 107.2648(19)°, *V* = 1863.64(7) Å^3, *Z* = 2, ρ_{calcd} = 1.597 Mg·m^{-3}, Cu-K_α radiation (*λ* = 1.54184 Å), *T* = 120 K, θ_{range} = 2.8-70.9°. Reflections collected 75311, independent reflections 7123, R_{int} = 0.0457. Final *R* indices [$F_o > 4\sigma(F_o)$]: R_1 = 0.0967, wR_2 = 0.2608.

{[B(μ-hpp)]$_2$(bpy)}$_2$(OTf)$_4$ (**4**(OTf)$_4$): Diborane ditriflate [TfOB(μ-hpp)]$_2$ (**1**) (113 mg, 0.190 mmol) and 4,4`-bipyridine (bpy) (32 mg, 0.205 mmol) were put in a dry nitrogen flushed Schlenk flask and dissolved in 4.5 mL of absolute degassed methylene chloride. The dark green reaction mixture turned orange upon stirring at room temperature overnight. Bright red crystals of the product were obtained by overlaying the methylene chloride solution with 4.5 mL of *n*-pentane. The crystals were collected and dried in vacuo yielding 45 mg (31% yield, 0.029 mmol) of **4**(OTf)$_4$ · 0.5 DCM.

C,H,N analysis (%) for $C_{52}H_{64}N_{16}B_4S_4O_{12}F_{12}$ · 0.5 DCM (1547.14 g mol^{-1}): calcd. C 40.76, H 4.23, N 14.49; found C 40.76, H 4.41, N 14.66. ^1H NMR (400 MHz, CD_3CN): *δ* = 8.21 (d, $^3J_{HH}$ = 6.9 Hz, 8H, H_{arom}), 7.82 (d, $^3J_{HH}$ = 7.0 Hz, 8H, H_{arom}), 3.47–3.28 (m, 32H, N-CH_2), 2.22–2.02 (m, 16H, CH_2) ppm. ^{11}B NMR (128 MHz, CD_3CN): *δ* = 3.90 (s, 4*B*) ppm. $^{11}B\{^1H\}$ NMR (128 MHz, CD_3CN): *δ* = 3.90 (s, 4*B*) ppm. ^1J CH-Korr (HSQC) NMR (400 MHz, CD_2Cl_2): *δ* = 144.7 (C_{arom}-H), 125.3 (C_{arom}-H), 46.9 (NCH_2), 40.1 (NCH_2), 21.0 (CH_2) ppm. ^{13}C NMR (101 MHz, CD_3CN): *δ* = 161.3 ($C_{gua,hpp}$), 145.7 (C_{arom}), 145.2 (C_{arom}-H), 125.9 (C_{arom}-H), 47.9 (NCH_2), 41.2 (NCH_2), 22.3 (CH_2) ppm. ^{19}F NMR (376 MHz, CD_3CN): *δ* = –79.17 ($SO_3CF_3^-$) ppm. MS (HR-ESI$^+$): m/z (%) = 603.2457 (**4**(OTf)$_2^{2+}$, 92%), 759.3147 ([B(μ-hpp)]$_2$(bpy)$_2$(OTf)$^+$, 100%), 1355.4485 (**4**(OTf)$_3^+$, 11%). IR (KBr): $\tilde{\nu}$ = 3237 (w), 3111 (w), 3029 (vw), 2948 (m, C_{hpp}-H), 2872 (m, C_{hpp}-H), 1623 (vs), 1545 (vs), 1488 (m), 1424 (m), 1399 (m), 1376 (w), 1326 (s), 1274 (s), 1225 (s), 1152 (s), 1101 (m), 1055 (m), 1029 (s), 974 (m), 913 (m), 872 (w), 843 (s), 745 (m), 702 (vw), 636 (vs), 593 (w), 572 (s), 517 (s), 482 (w) cm^{-1}.

{[B(μ-hpp)]$_2$(bpy)}$_2$[B(C$_6$F$_5$)$_4$]$_4$ (**4**[B(C$_6$F$_5$)$_4$]$_4$): In a dry argon flushed Schlenk flask compound **4**(OTf)$_4$ (20 mg, 0.013 mmol) was dissolved in 2 mL of absolute methylene chloride and a suspension of sodium tetrakis(pentafluorophenyl)borate (39 mg, 0.056 mmol) in 3 mL of methylene chloride was added. The reaction mixture was stirred overnight at room temperature and filtrated under inert conditions. The orange filtrate was overlayered with 5 mL of absolute *n*-pentane whereby an orange oil formed. The supernatant was removed via syringe and the residue dried in vacuo to yield 19% (9 mg, 0.002 mmol) of product. Crystals suitable for an X-ray analysis were obtained by diffusion of *n*-pentane into a saturated methylene chloride solution of **4**[B(C$_6$F$_5$)$_4$]$_4$ at –18 °C.

C,H,N analysis (%) for $C_{144}H_{64}N_{16}B_8F_{80}$ · DCM (3709.49 g mol^{-1}): calcd. C 46.95, H 1.79, N 6.04; found C 46.77, H 2.29, N 6.14. ^1H NMR (400 MHz, CD_2Cl_2): *δ* = 8.00 (d, $^3J_{HH}$ = 6.7 Hz, 8H, C_{arom}-*H*), 7.44 (d, $^3J_{HH}$ = 6.9 Hz, 8H, C_{arom}-*H*), 3.57–3.25 (m, 32H, N-CH_2), 2.28–2.02 (m, 16H, CH_2) ppm. ^{11}B NMR (128 MHz, CD_2Cl_2): *δ* = –16.71 (s, sharp, 4B, [*B*(C$_6$F$_5$)$_4$]$^-$) ppm, *signal for the cation was not visible*. ^{11}B NMR (128 MHz, CD_3CN): *δ* = 4.17 (s, broad, 4*B*), –16.71 (s, sharp, 4B, [*B*(C$_6$F$_5$)$_4$]$^-$) ppm. ^1J CH-Korr (HSQC) NMR (400 MHz, CD_2Cl_2): *δ* = 143.7 (C_{arom}-H), 124.2 (C_{arom}-H), 47.3 (NCH_2), 21.1 (CH_2) ppm. $^{13}C\{^1H\}$ NMR (101 MHz, CD_2Cl_2): *δ* = 160.8 ($C_{gua,hpp}$), 148.7 (dm, *o*-*C*F, $^1J_{13C-19F}$ = 255 Hz), 145.1 ($C_{q,arom}$), 144.0 (C_{arom}-H), 138.7 (dm, *p*-*C*F, $^1J_{13C-19F}$ = 245 Hz), 136.8 (dm, *m*-*C*F, $^1J_{13C-19F}$ = 244 Hz), 124.8 (C_{arom}-H), 124.2 (br, C_{ipso}), 48.0 (NCH_2), 41.3 (NCH_2), 22.0 (CH_2) ppm. ^{19}F NMR (376 MHz, CD_2Cl_2): *δ* = –132.99 (br, *o*-C*F*), –163.07 (t, $^3J_{FF}$ = 20.5 Hz, *p*-C*F*), –167.20 (pt, $^3J_{FF}$ = 17.6 Hz, *m*-C*F*) ppm. MS (HR-ESI$^+$): m/z (%) = 1133.2761 (**4**[B(C$_6$F$_5$)$_4$]$_2^{2+}$, 39%). IR (KBr): $\tilde{\nu}$ = 2964 (vw, C_{hpp}-H), 2881 (vw, C_{hpp}-H), 1704 (vw), 1644 (m), 1620 (m), 1540 (m), 1514 (s), 1464 (vs), 1375 (w), 1325 (m), 1271 (m), 1230 (w), 1189 (vw), 1089 (s), 1043 (w), 979 (vs), 910 (vw), 836 (w), 772 (w), 756 (m), 684 (m), 662 (m), 609 (vw), 573 (vw), 480 (vw) cm^{-1}. Crystal data for **4**[B(C$_6$F$_5$)$_4$]$_4$ · 4 DCM, M_r = 3964.29, 0.14 x 0.05 x 0.01 mm, triclinic, space group P–1 (*IT* Nr. 2), *a* = 15.9582(6), *b* = 21.1543(10), *c* = 23.9381(6) Å, *α* = 101.843(3)°, *β* = 95.880(3)°, *γ* = 105.077(4)°, *V* = 7532.3(5) Å^3, *Z* = 2, ρ_{calcd} = 1.748 Mg·m^{-3}, Cu-K_α radiation (*λ* = 1.54184 Å), *T* = 120 K, θ_{range} = 2.2-68.0°. Reflections collected 159375, independent reflections 27000, R_{int} = 0.1553. Final *R* indices [$F_o > 4\sigma(F_o)$]: R_1 = 0.0678, wR_2 = 0.1507.

{[B(μ-hpp)]$_2$(dpe)}$_2$(OTf)$_4$ (**5**(OTf)$_4$): Diborane ditriflate [TfOB(μ-hpp)]$_2$ (**1**) (25 mg, 0.042 mmol) was put in a dry argon flushed Schlenk flask and dissolved in 1 mL of absolute degassed methylene chloride. A solution of 1,2-di(4-pyridyl)ethylene (dpe) (8 mg, 0.046 mmol) dissolved in 1 mL absolute degassed methylene chloride was added at room temperature and the reaction mixture turned immediately red. The reaction mixture was stirred for 3 h at room temperature. The solution was filtered, and the filtrate was overlayered with 2 mL of *n*-pentane under formation of a red oil. The colorless supernatant was removed via syringe and the oil was dissolved in 2 mL of methylene chloride. Deep red crystals were obtained by overlaying the solution with 1 mL of *n*-pentane. The crystals were collected and dried in vacuo yielding 7 mg (23%, 0.005 mmol) of product.
^{1}H NMR (400 MHz, CD$_3$CN): δ = 7.98 (d, $^{3}J_{HH}$ = 6.8 Hz, 8H, H$_{arom}$), 7.57 (d, $^{3}J_{HH}$ = 6.4 Hz, 8H, H$_{arom}$), 7.33 (s, 4H, CH), 3.45-3.26 (m, 32H, N-CH$_2$), 2.19-2.01 (m, 16H, CH$_2$) ppm. ^{11}B NMR (128 MHz, CD$_3$CN): δ = 3.16 (s, 4*B*) ppm. ^{11}B{^{1}H} NMR (128 MHz, CD$_3$CN): δ = 3.16 (s, 4*B*) ppm. ^{13}C NMR (101 MHz, CD$_3$CN): δ = 160.9 ($C_{gua,hpp}$), 147.3 (C_{arom}), 143.9 (C_{arom}), 132.4 (C$_{arom}$), 125.2 (*C*H), 47.6 (N*C*H$_2$), 40.8 (N*C*H$_2$), 22.0 (*C*H$_2$) ppm. ^{19}F NMR (376 MHz, CD$_3$CN): δ = –79.21 (SO$_3$C*F*$_3^-$) ppm. MS (HR-ESI$^+$): m/z (%) = 369.5248 (**5**(OTf)$^{3+}$, 7%), 628.7638 (**5**(OTf)$_2^{2+}$, 100%), 1407.4826 (**5**(OTf)$_3^+$, 64%). IR (KBr): $\tilde{\nu}$ = 3558 (w), 3490 (w), 3112 (w), 2959 (m), 2871 (m), 1624 (s), 1608 (s), 1546 (s), 1508 (m), 1442 (m), 1325 (m), 1277 (s), 1262 (s), 1226 (s), 1154 (s), 1055 (m), 1030 (s), 976 (m), 916 (m), 861 (m), 839 (w), 752 (m), 638 (s), 573 (w), 518 (m) cm^{-1}.

{[B(μ-hpp)]$_2$(dpe)}$_2$[B(C$_6$F$_5$)$_4$]$_4$ (**5**[B(C$_6$F$_5$)$_4$]$_4$): In a dry argon flushed Schlenk flask compound **5**(OTf)$_4$ (12 mg, 0.008 mmol) was dissolved in 1 mL of absolute methylene chloride and a suspension of sodium tetrakis(pentafluorophenyl)borate (24 mg, 0.035 mmol) in 3 mL of methylene chloride was added. The reaction mixture was stirred overnight at room temperature and filtrated under inert conditions. The orange filtrate was overlayered with 3 mL of absolute *n*-pentane whereby an orange oil formed. It was stored at –30°C to form crystals suitable for an X-ray analysis.
Crystal data for **5**[B(C$_6$F$_5$)$_4$]$_4$ · 2 DCM, M_r = 3846.51, 0.27 x 0.20 x 0.12 mm, monoclinic, space group $P2_1/n$ (*IT* Nr. 14), a = 19.4647(7), b = 16.0608(5), c = 25.4409(9) Å, $\alpha = \gamma$ = 90°, β = 104.883(2)°, V = 7686.5(5) Å^3, Z = 2, ρ_{cald} = 1.662 Mg·m^{-3}, Cu-K_α radiation (λ = 1.54178 Å), T = 120 K, θ_{range} = 2.6-66.5°. Reflections collected 74973, independent reflections 6809, R_{int} = 0.1071. Final R indices [$F_o > 4\sigma(F_o)$]: R_1 = 0.0864, wR_2 = 0.2618.

{[B(μ-hpp)]$_2$(daz)OTf}$_2$(OTf)$_2$ (**6**(OTf)$_2$): In a dry argon flushed Schlenk flask compound **1** [TfOB(μ-hpp)]$_2$ (25 mg, 0.042 mmol) and 1,4-diazine (4 mg, 0.046 mmol) were dissolved in 3.5 mL of absolute degassed methylene chloride. The deep red solution was stirred at room temperature for 1.5 hours and subsequently overlayered with 3.5 mL of absolute pentane. The orange product crystallized directly from the reaction mixture in 17% yield (5 mg, 0.003 mmol).
C,H,N analysis (%) for $C_{40}H_{56}N_{16}B_4S_4O_{12}F_{12}$ · DCM (1437.41 g mol^{-1}): calcd. C 34.26, H 4.07, N 15.59; found C 33.71, H 4.34, N 15.67. MS (HR-ESI$^+$): m/z (%) = 527.2147 (**6**$^{2+}$, 30%), 1203.3832 (**6**(OTf)$^+$, 100%). IR (KBr): $\tilde{\nu}$ = 3245 (w), 2963 (m, C$_{hpp}$-H), 2879 (w, C$_{hpp}$-H), 1633 (s), 1597 (vs), 1545 (vs), 1461 (m), 1404 (m), 1381 (s), 1325 (s), 1262 (s), 1211 (s), 1161 (vs), 1089 (s), 1030 (vs), 970 (s), 876 (w), 860 (w), 802 (m), 756 (vw), 731 (vw), 708 (vw), 638 (vs), 573 (w), 518 (s), 483 (w) cm^{-1}. Crystal data for **6**(OTf)$_2$, M_r = 1352.48, 0.20 x 0.12 x 0.04 mm, monoclinic, space group $C2/c$ (*IT* Nr. 15), a = 27.3348(6), b = 11.3026(3), c = 17.6983(3) Å, $\alpha = \gamma$ = 90°, β = 95.5992(18)°, V = 5441.9(2) Å^3, Z = 4, ρ_{cald} = 1.651 Mg·m^{-3}, Cu-K_α radiation (λ = 1.54184 Å), T = 120 K, θ_{range} = 3.2-71.2°. Reflections collected 98856, independent reflections 5216, R_{int} = 0.0742. Final R indices [$F_o > 4\sigma(F_o)$]: R_1 = 0.0707, wR_2 = 0.1846.

{[B(μ-hpp)]$_2$(dpp)}(OTf)$_2$ (**7**(OTf)$_2$): Diborane ditriflate [TfOB(μ-hpp)]$_2$ (**1**) (25 mg, 0.042 mmol) and 2,3-di-2-pyridinyl-pyrazine (dpp) (10 mg, 0.044 mmol) were dissolved in 3.5 mL of absolute degassed methylene chloride. The dark red reaction mixture was stirred for 1.5 h at room temperature and subsequently overlayered with 3.5 mL of absolute degassed *n*-pentane. After a few hours an oily brown residue formed and turned to pale yellow crystals after one week. The solvent and remaining oily residue were removed via decanting and the solid was dried in vacuo. Due to varying amounts of the crystalline product we determined the reaction yield (70%) by means of ^{1}H NMR spectroscopy.
C,H,N analysis (%) for $C_{30}H_{34}N_{10}B_2O_6S_2F_6$ · 0.5 DCM (872.87 g mol^{-1}): calcd. C 41.97, H 4.04, N 16.05; found C 42.02, H 4.13, N 16.38. ^{1}H NMR (400 MHz, CD$_2$Cl$_2$): δ = 8.93 (s, 2H, C$_{arom}$-*H*), 8.42 (d, $^{3}J_{HH}$ = 5.5 Hz, 2H, C$_{arom}$-*H*), 8.18 (dd, $^{3}J_{HH}$ = 7.8 Hz, $^{4}J_{HH}$ = 1.3 Hz, 2H, C$_{arom}$-*H*), 7.88 (ddd, $^{3}J_{HH}$ = 7.6 Hz, $^{3}J_{HH}$ = 6.1 Hz, $^{4}J_{HH}$ = 1.4 Hz, 2H, C$_{arom}$-*H*), 7.63 (dd, $^{3}J_{HH}$ = 7.8 Hz, $^{4}J_{HH}$ = 0.9 Hz, 2H, C$_{arom}$-*H*), 3.55–3.27 (m, 8H, N-C*H*$_2$), 3.18–3.08 (m, 4H, N-C*H*$_2$), 2.86–2.71 (m, 4H, N-C*H*$_2$), 2.35–2.24 (m, 2H, C*H*$_2$), 2.12–2.07 (m, 2H, C*H*$_2$), 1.85–1.65 (m, 4H, C*H*$_2$) ppm. ^{11}B NMR (128 MHz, CD$_2$Cl$_2$): δ = 3.44 (s, 2B) ppm. ^{11}B{^{1}H} NMR (128 MHz, CD$_2$Cl$_2$): δ = 3.46 (s, 2B) ppm. ^{13}C NMR (101 MHz, CD$_2$Cl$_2$): δ = 162.2 (C_q, hpp), 158.3 (C_q, hpp), 150.6 (C_{arom}), 147.7 (C_{arom}), 145.7 (C_{arom}-H), 145.2 (C_{arom}-H), 143.3 (C_{arom}-H), 131.0 (C_{arom}-H), 128.7 (C_{arom}-H), 121.5 (q, SO$_3$C*F*$_3^-$), 48.0 (N*C*H$_2$), 46.9 (N*C*H$_2$), 41.4 (N*C*H$_2$), 41.0 (N*C*H$_2$), 21.9 (*C*H$_2$), 20.6 (*C*H$_2$) ppm. ^{19}F NMR (376 MHz, CD$_2$Cl$_2$): δ = –78.81 (SO$_3$C*F*$_3^-$) ppm. MS (HR-ESI$^+$): m/z (%) = 681.2668 (**7**(OTf)$^+$, 100%). IR (KBr): $\tilde{\nu}$ = 3136 (vw), 3076 (vw), 2954 (w), 2871 (w), 1613 (s), 1571 (w), 1541 (s), 1491 (vw), 1448 (w), 1395 (w), 1329 (m), 1266 (s), 1222 (m), 1191 (w), 1152 (s), 1107 (w), 1096 (w), 1057 (w), 1029 (vs), 962 (w), 916 (w), 865 (w), 845 (w), 811 (vw), 782 (m), 753 (w), 726 (vw), 708 (vw), 637 (vs), 600 (w), 572 (w), 517 (m) 489 (vw), 448 (vw) cm^{-1}. Crystal data for **7**(OTf)$_2$ · DCM, M_r = 915.34, 0.5 x 0.4 x 0.3 mm, monoclinic, space group $P2_1/m$ (*IT* Nr. 11), a = 9.922(2), b = 13.448(3), c = 14.782(3) Å, $\alpha = \gamma$ = 90°, β = 106.75(3)°, V = 1888.7(7) Å^3, Z = 2, ρ_{cald} = 1.610 Mg·m^{-3}, Mo-K_α radiation (λ = 0.71073 Å), T = 120 K, θ_{range} = 1.8-48.0°. Reflections collected 5740, independent reflections 3899, R_{int} = 0.0583. Final R indices [$F_o > 4\sigma(F_o)$]: R_1 = 0.0578, wR_2 = 0.1700.

Analytical Data

Analytical data for **2**(OTf)$_2$.

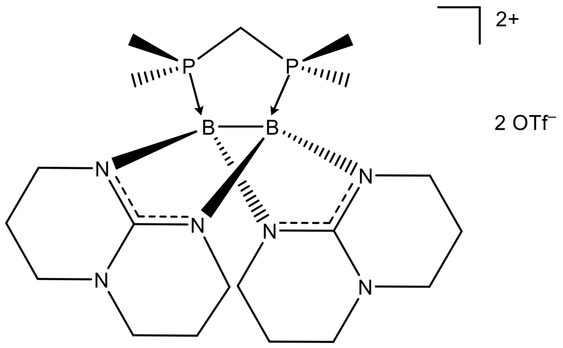

2

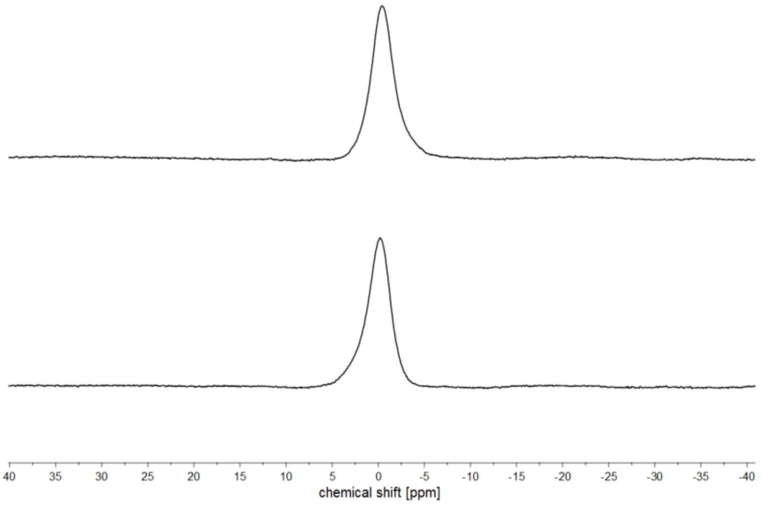

Figure S1. $^{11}B\{^{1}H\}$ NMR (128 MHz, CD_2Cl_2, top) and ^{11}B NMR (128 MHz, CD_2Cl_2, bottom) spectra of **2**(OTf)$_2$.

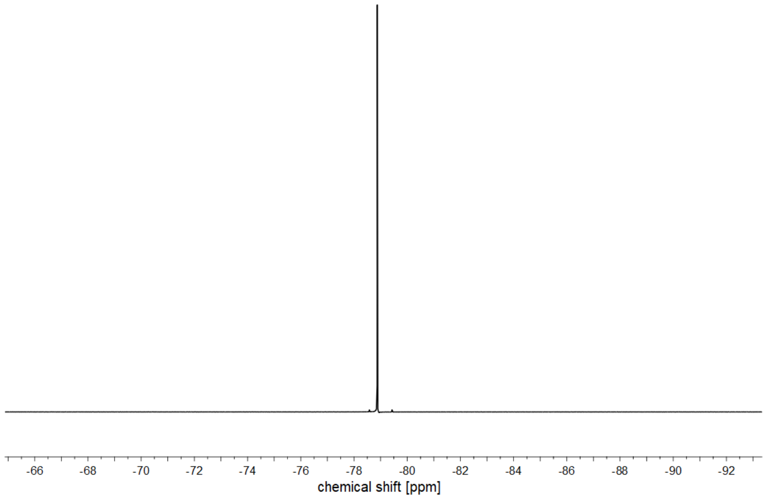

Figure S2. ^{19}F NMR (376 MHz, CD_2Cl_2) spectrum of **2**(OTf)$_2$.

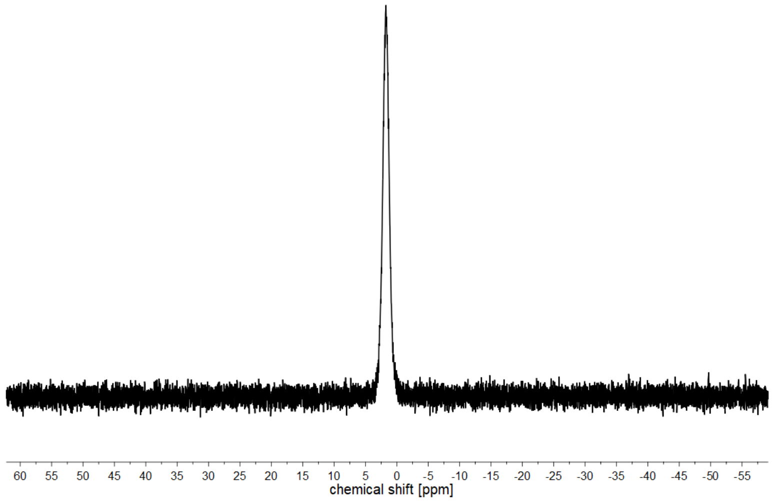

Figure S3. ^{31}P NMR (162 MHz, CD_2Cl_2) spectrum of **2**$(OTf)_2$.

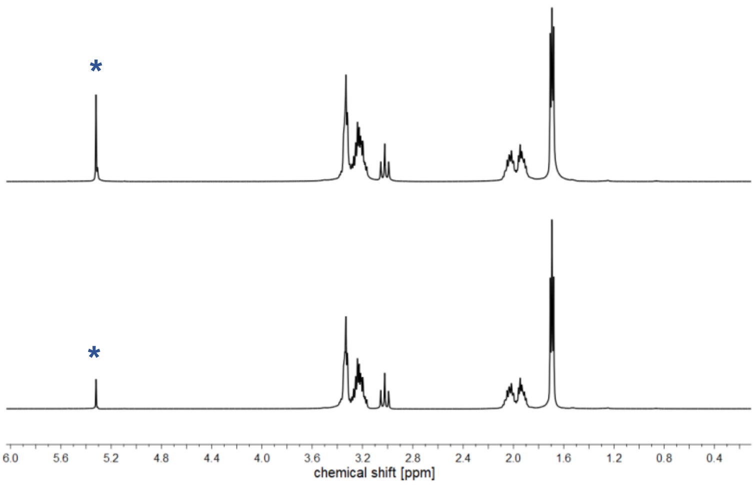

Figure S4. ^{1}H NMR (400 MHz, CD_2Cl_2, top) and $^{1}H\{^{11}B\}$ NMR (400 MHz, CD_2Cl_2, bottom) spectra of **2**$(OTf)_2$. The peaks marked by an asterisk denote the solvent residual signal of CH_2Cl_2.

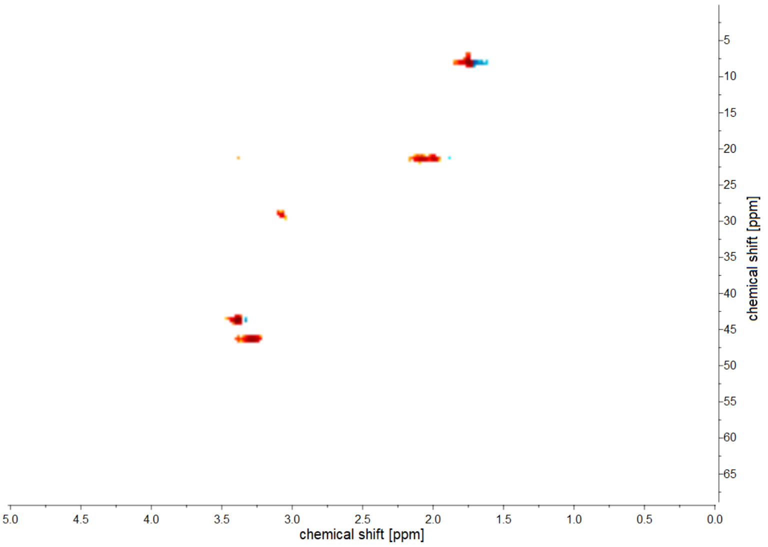

Figure S5. ^{1}J CH-Korr (HSQC) NMR (400 MHz, CD_2Cl_2) spectrum of **2**$(OTf)_2$.

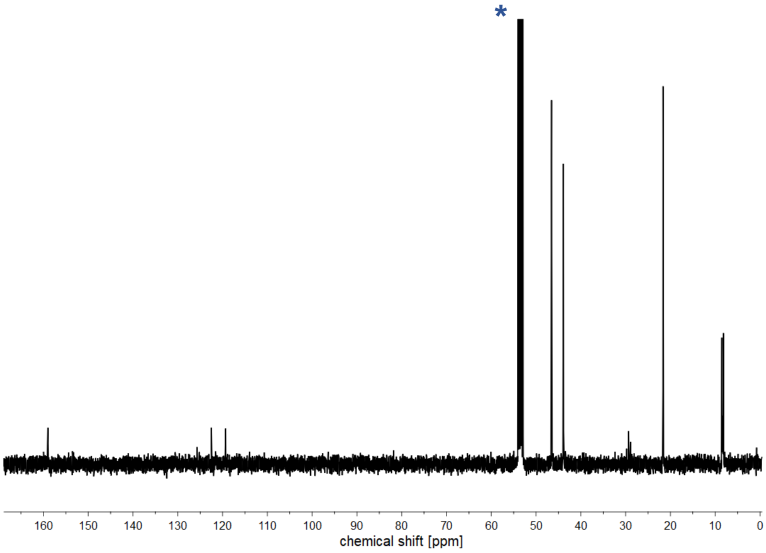

Figure S6. $^{31}C\{^{1}H\}$ NMR (101 MHz, CD_2Cl_2) spectrum of **2**$(OTf)_2$. The peak marked by an asterisk denotes the solvent residual signal of CH_2Cl_2.

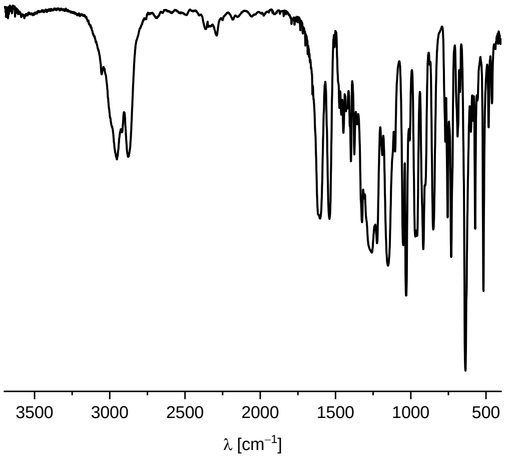

Figure S7. IR spectrum of **2**$(OTf)_2$ as a KBr disk.

Analytical data for **3**(OTf)

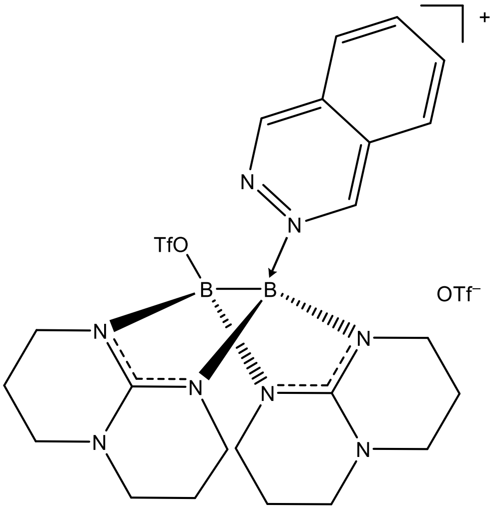

3

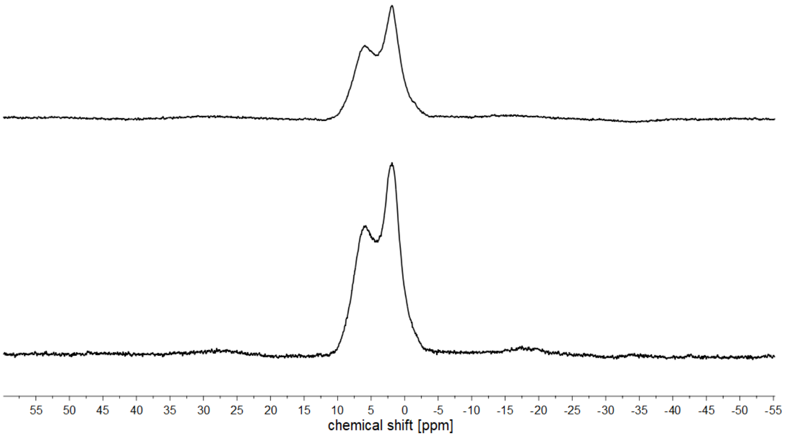

Figure S8. ^{11}B NMR (128 MHz, CD_2Cl_2, top) and $^{11}B\{^1H\}$ NMR (128 MHz, CD_2Cl_2, bottom) spectra of **3**(OTf).

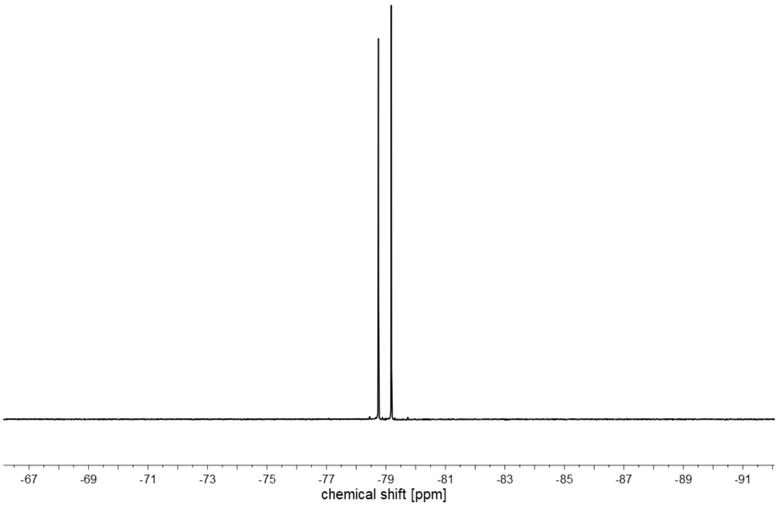

Figure S9. ^{19}F NMR (376 MHz, CD_2Cl_2) spectrum of **3**(OTf).

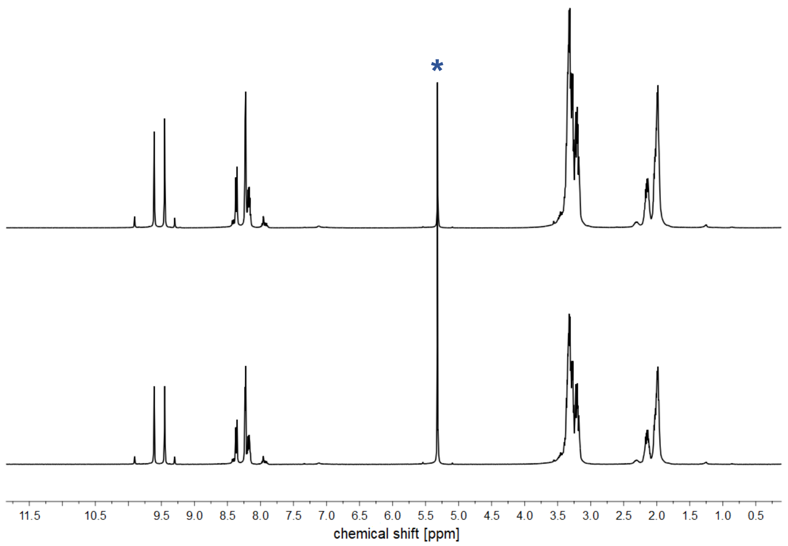

Figure S10. $^{1}H\{^{11}B\}$ NMR (400 MHz, CD_2Cl_2, top) and ^{1}H NMR (400 MHz, CD_2Cl_2, bottom) spectra of **3**(OTf). The peak marked by an asterisk denotes the solvent residual signal of CH_2Cl_2.

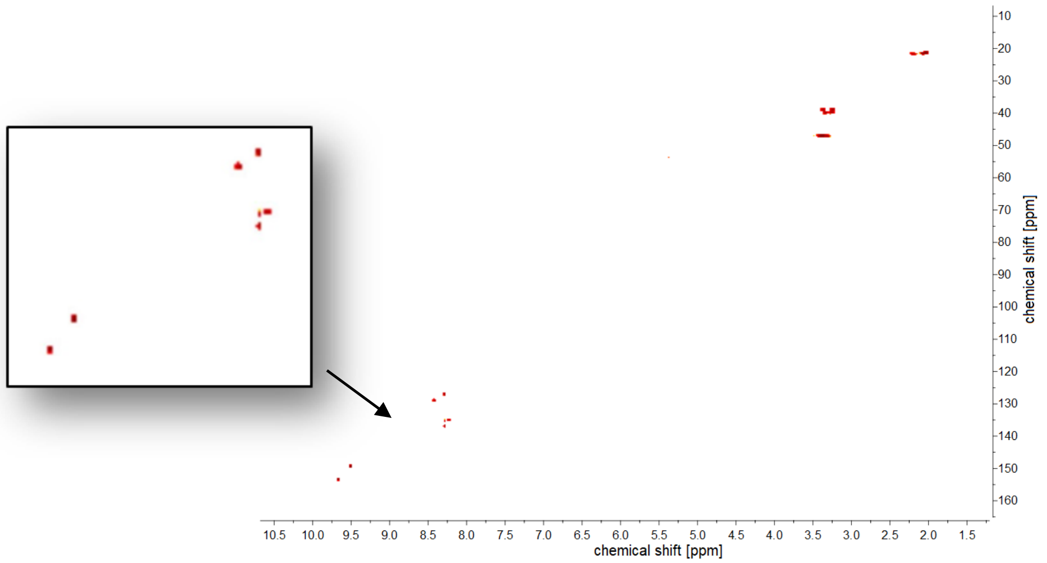

Figure S11. ^{1}J CH-Korr (HSQC) NMR (400 MHz, CD_2Cl_2) spectrum of **3**(OTf).

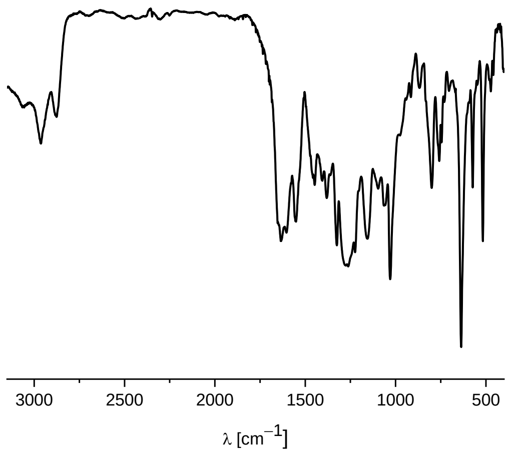

Figure S12. IR spectrum of **3**(OTf) as a KBr disk.

Analytical data for **4**(OTf)$_4$

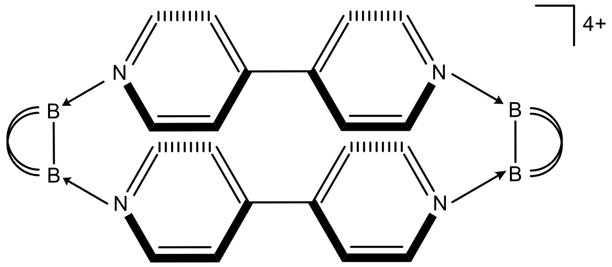

4 4 OTf⁻

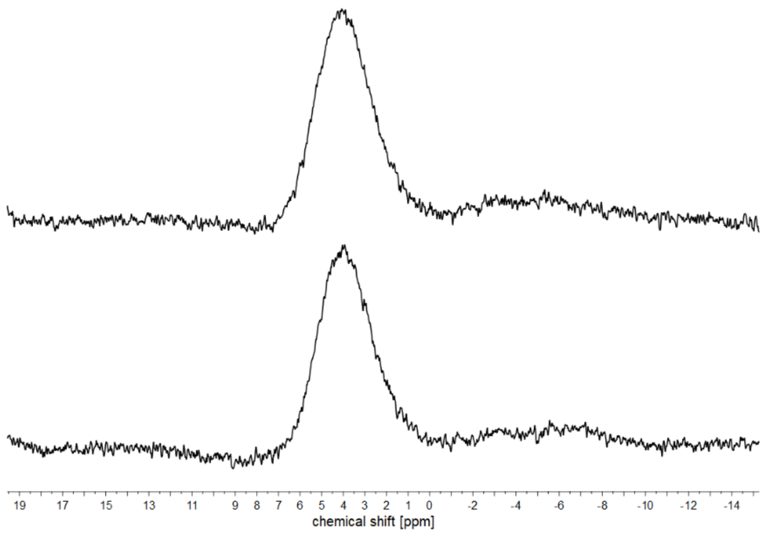

Figure S13. ^{11}B NMR (128 MHz, CD_3CN, top) and $^{11}B\{^1H\}$ NMR (128 MHz, CD_3CN, bottom) spectra of **4**(OTf)$_4$.

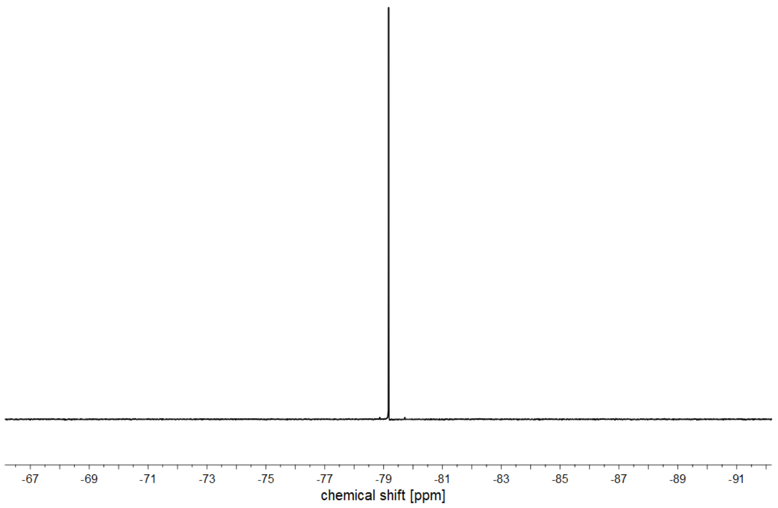

Figure S14. ^{19}F NMR (376 MHz, CD_3CN) spectrum of **4**(OTf)$_4$.

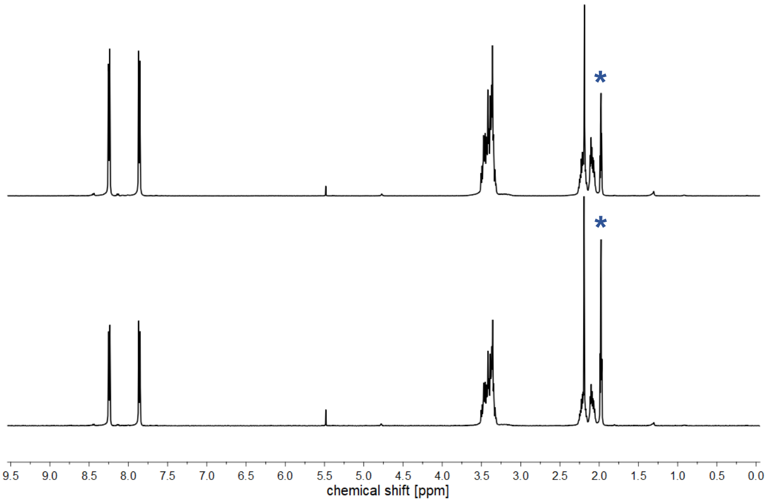

Figure S15. $^{1}H\{^{11}B\}$ NMR (400 MHz, CD_3CN, top) and ^{1}H NMR (400 MHz, CD_3CN, bottom) spectra of **4**(OTf)$_4$. The peaks marked by an asterisk denote the solvent residual signal of CH_3CN.

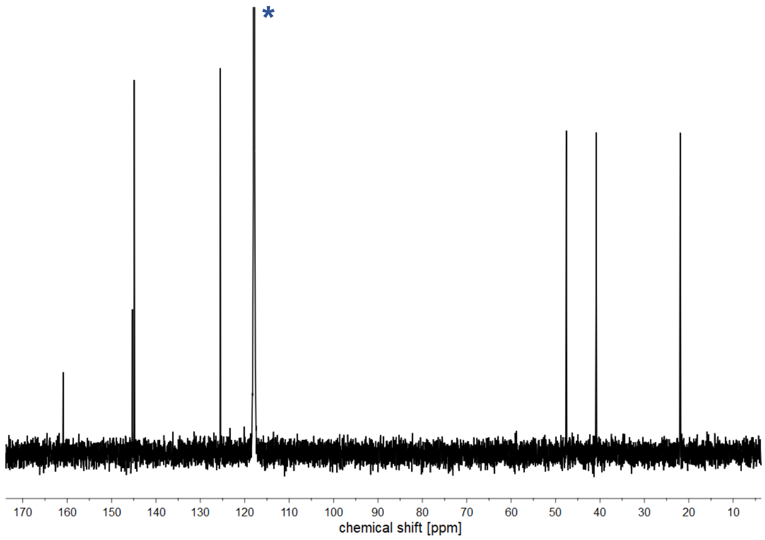

Figure S16. $^{31}C\{^{1}H\}$ NMR (101 MHz, CD_3CN) spectrum of **4**(OTf)$_4$. The peak marked by an asterisk denotes the solvent residual signal of CD_3CN.

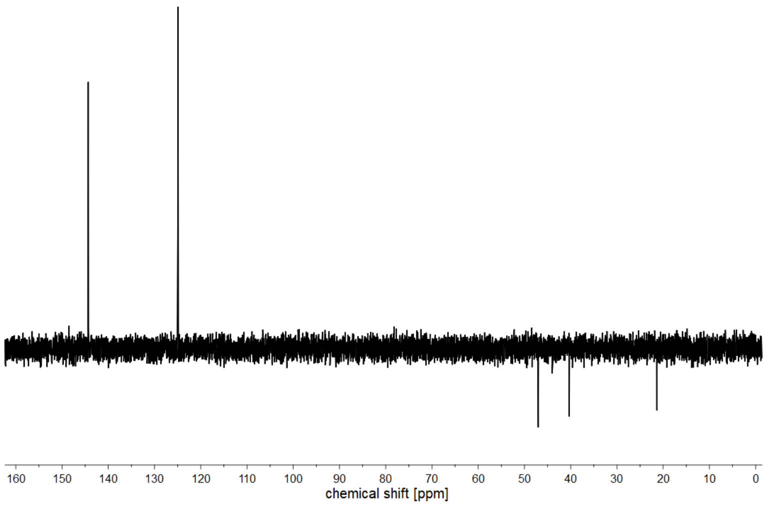

Figure S17. $^{31}C\{^{1}H\}$ DEPT135 NMR (101 MHz, CD_3CN) spectrum of **4**(OTf)$_4$.

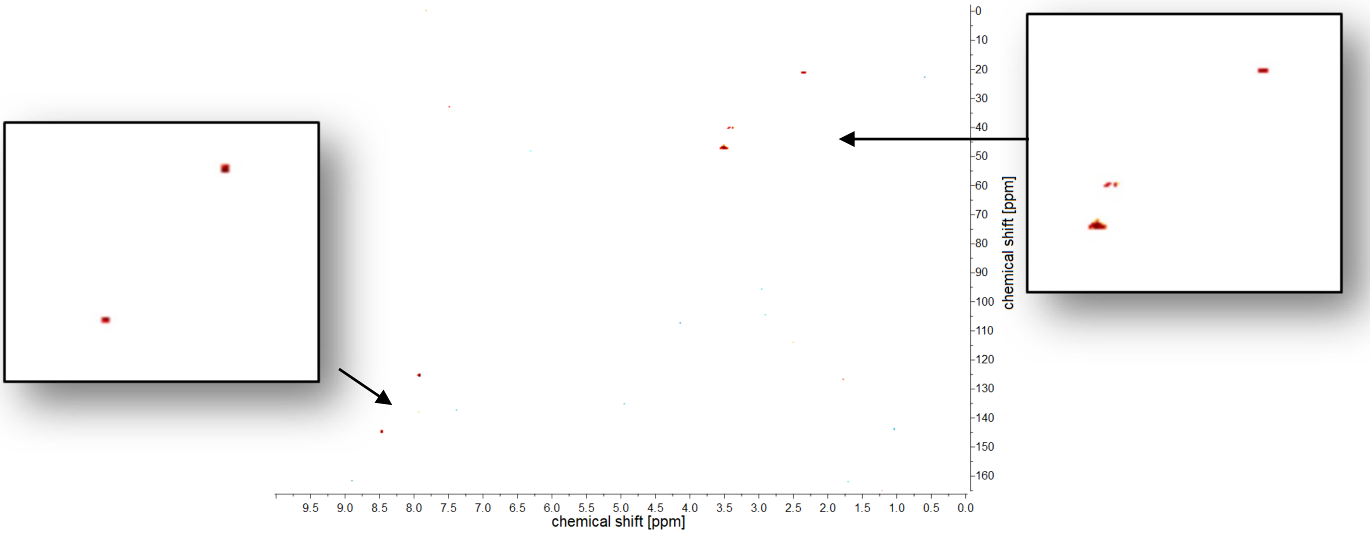

Figure S18. [1]J CH-Korr (HSQC) NMR (400 MHz, CD_2Cl_2) spectrum of **4**(OTf)$_4$.

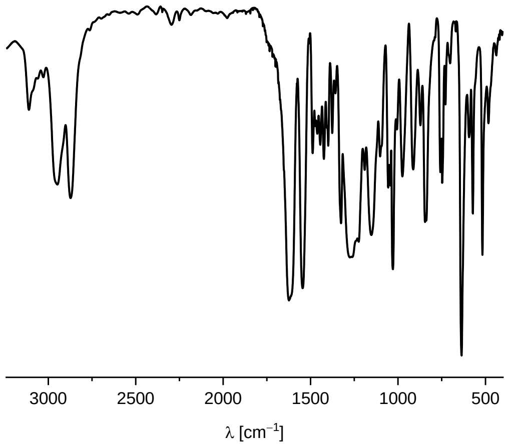

Figure S19. IR spectrum of **4**(OTf)$_4$ as a KBr disk.

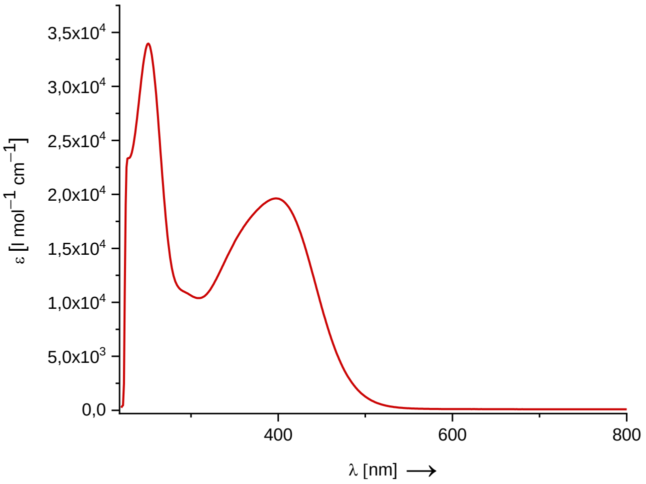

Figure S20. UV/Vis spectrum of **4**(OTf)$_4$ in CH_2Cl_2 at room temperature; c = 3.0 · 10^{-5} mol l^{-1}; λ_{max}/nm (ε in l mol^{-1} cm^{-1}) = 397 (2.0 · 10^4), 251 (3.4 · 10^4).

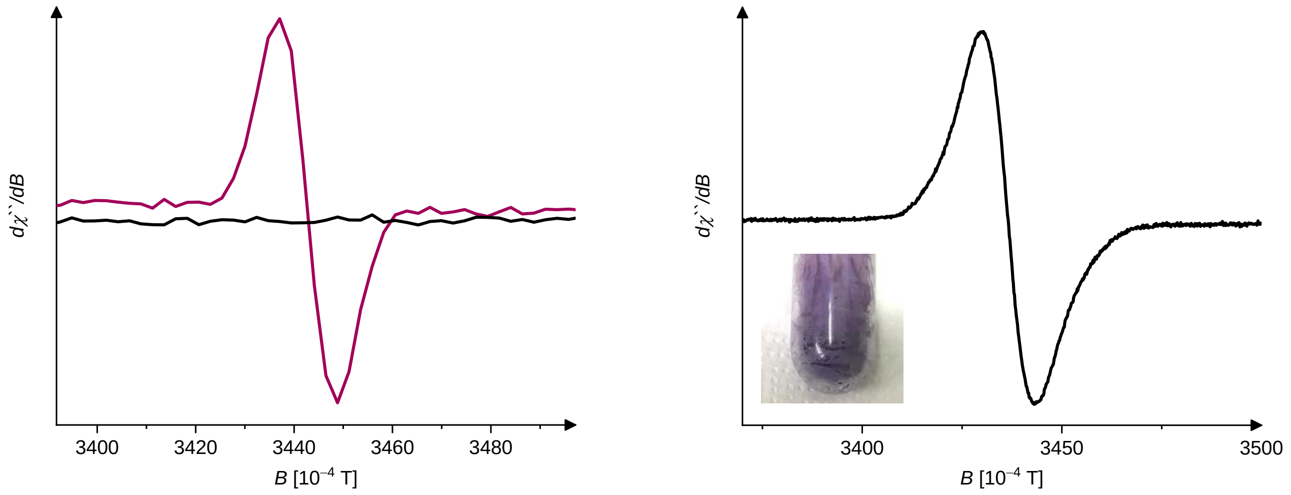

Figure S21. Left: EPR spectrum (X-band, ~9.63 GHz, CH_2Cl_2 solution at room temperature) of the reaction mixture under inert gas (red curve) and after stirring for 30 min at air exposure (black curve). Right: EPR spectrum (X-band, ~9.63 GHz, solid at room temperature) of the violet reaction product (in the photo) obtained by the reaction in THF under inert gas.

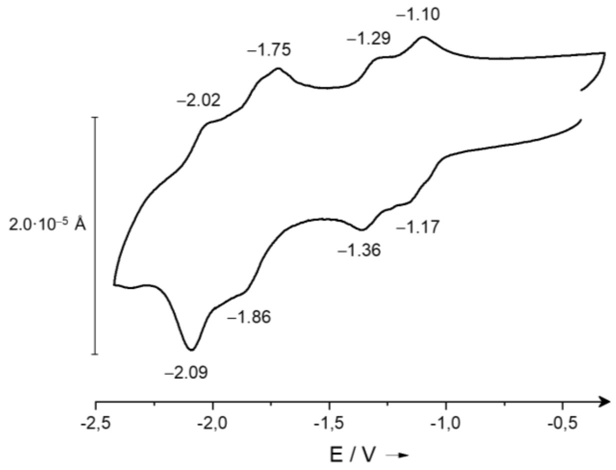

Figure S22. The cyclovoltamogramm of **4**(OTf)₄. A glassy carbon working electrode, a platinum counter electrode and an Ag/AgCl reference electrode were used to characterize a solution in CH_3CN at 298 K with 0.1 M $TBAPF_6$ serving as the electrolyte. A scan rate of 100 mV s^{-1} was used for this analysis and the obtained values were referenced to the ferrocenium/ferrocene (Fc^+/Fc) redox pair.

a)

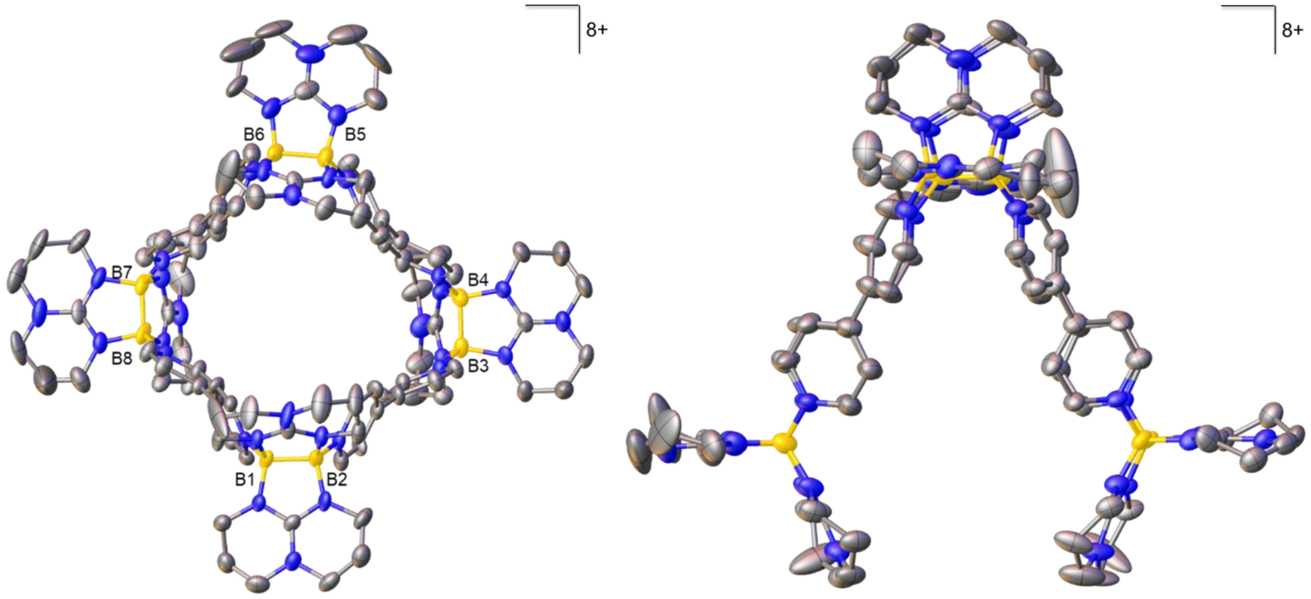

b)

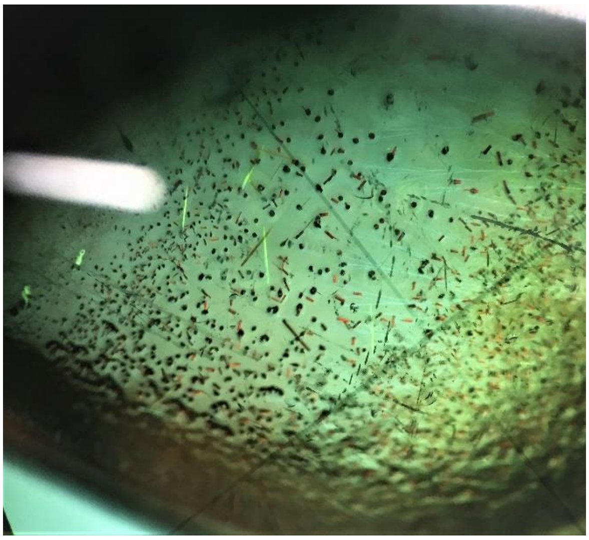

Figure S23. a) Illustration of the experimentally determined structure of the octacationic octaborane byproduct (top and side view). All hydrogen atoms and the eight OTf⁻ counterions are omitted for clarity. Displacement ellipsoids are drawn at the 50% probability level. Intensity data were obtained from two different crystals with the BRUKER *D8 Venture* (*T* = 100 and 120 K, Mo-K_α radiation, microfocus X-ray tubes, PHOTON III detector). Both datasets are characterized by a steep decrease of intensity with increasing resolution. The data were indexed in a triclinic unit cell in the space group *P*-1 (*Z* = 2). Not surprisingly in view of the poor data quality, refinement did not lead to satisfactory agreement factors for both datasets. Obviously, the difficulties in solving the structure are caused by severe disorder of the triflate anions, the conformational flexibility of the hpp rings and residual electron density within the macrocycle. The latter is possibly attributed to several H_2O molecules. However, the gross composition of the material and constitution of the cation(s) was clearly established. b) Photo of the crystals formed from the mother liquor showing the two different crystal types of **4**$(OTf)_4$ (red) and the octacationic species (yellow).

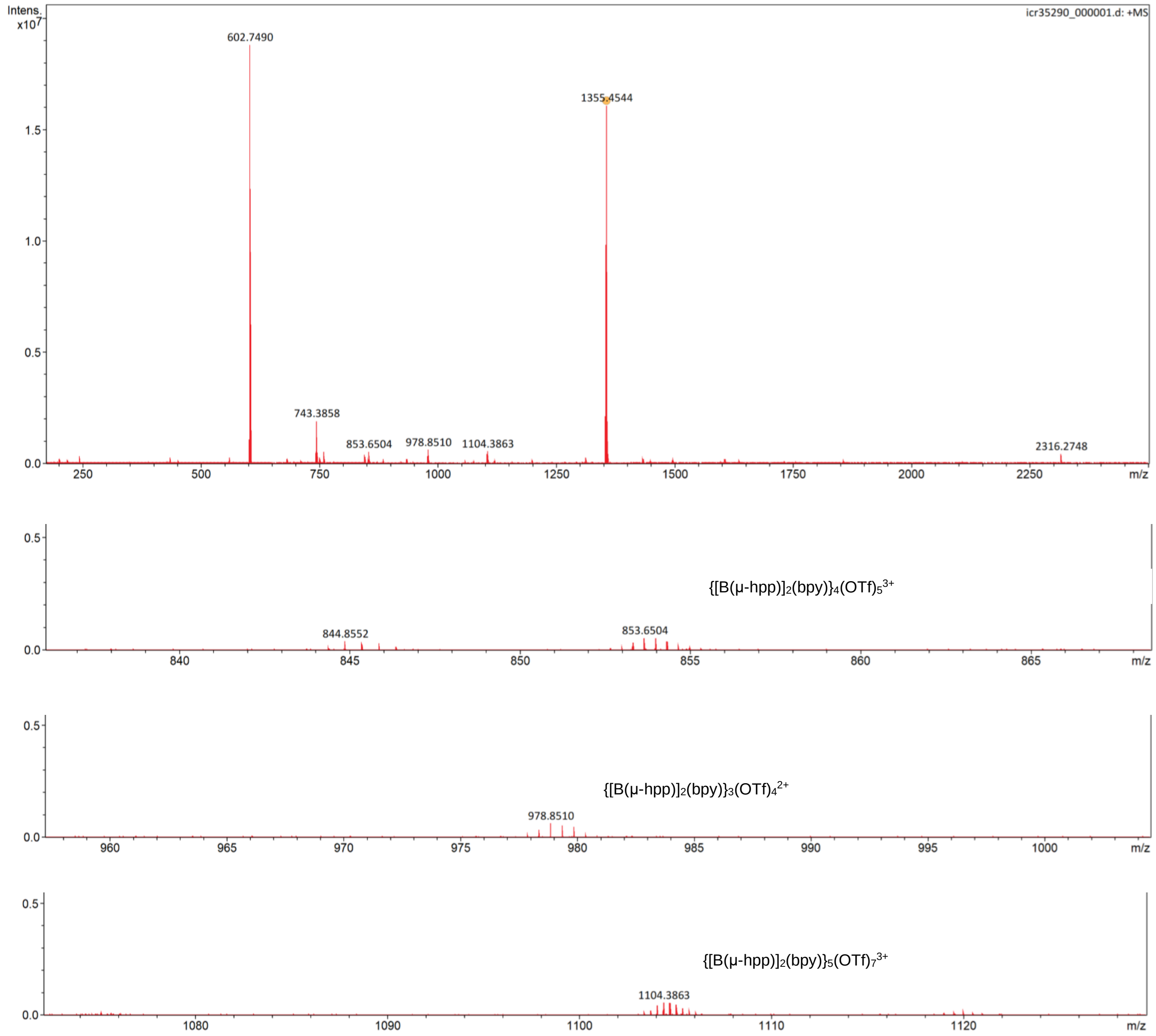

Figure S24. HR-ESI$^+$ mass spectrum of the reaction mixture of **4**(OTf)$_4$ recorded after heating in a CH_2Cl_2 solution.

Analytical data for **4**[B(C_6F_5)$_4$]$_4$

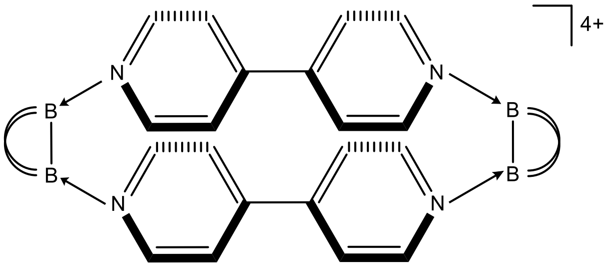

4 4 [B(C_6F_5)$_4$]$^-$

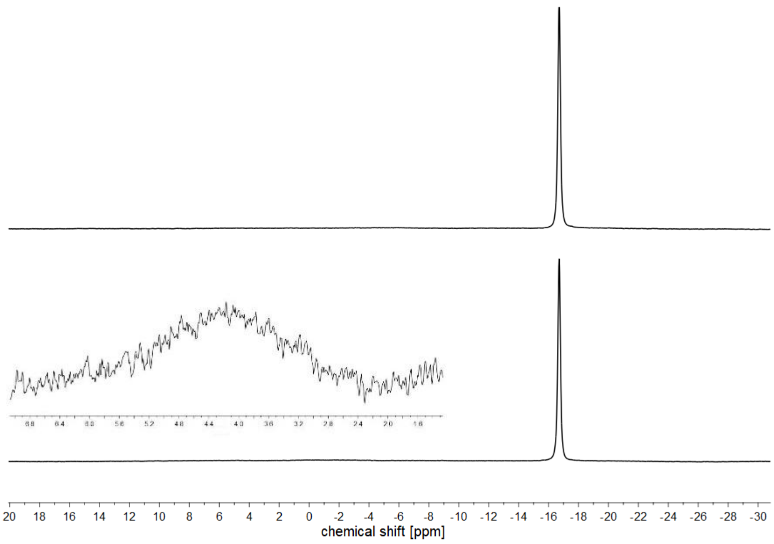

Figure S25. ^{11}B{^{1}H} NMR (128 MHz, CD_2Cl_2, top) and ^{11}B NMR (128 MHz, CD_2Cl_2, bottom) spectra of **4**[B(C_6F_5)$_4$]$_4$ together with the magnified region of the strongly broadened ^{11}B signal as observed in CD_3CN.

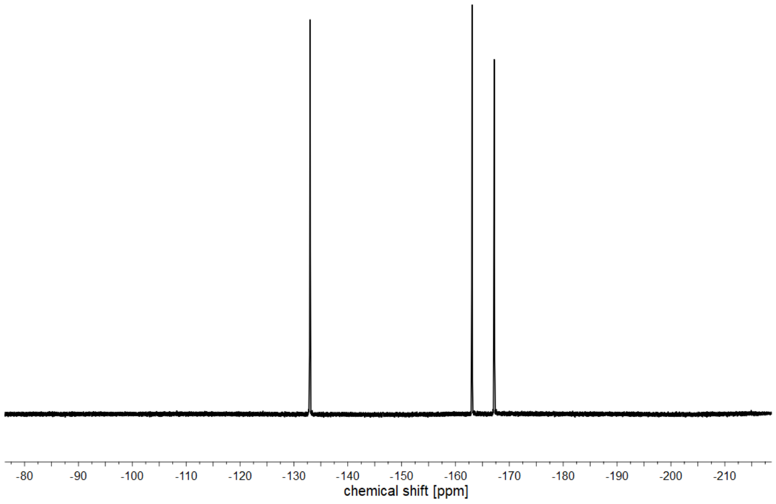

Figure S26. ^{19}F NMR (376 MHz, CD_2Cl_2) spectrum of **4**[B(C_6F_5)$_4$]$_4$.

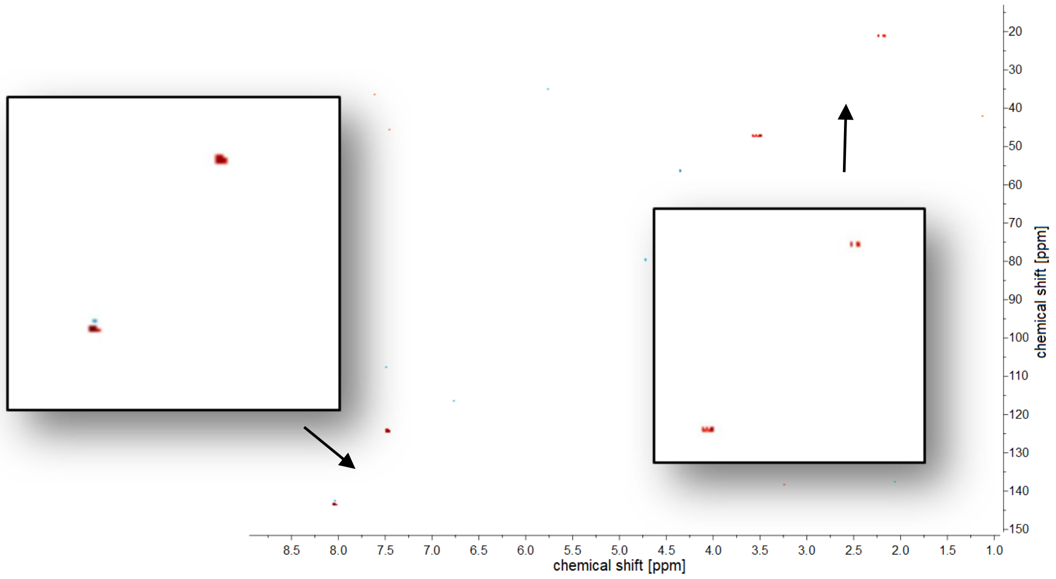

Figure S27. [1]J CH-Korr (HSQC) NMR (400 MHz, CD_2Cl_2) spectrum of **4**[B(C_6F_5)$_4$]$_4$.

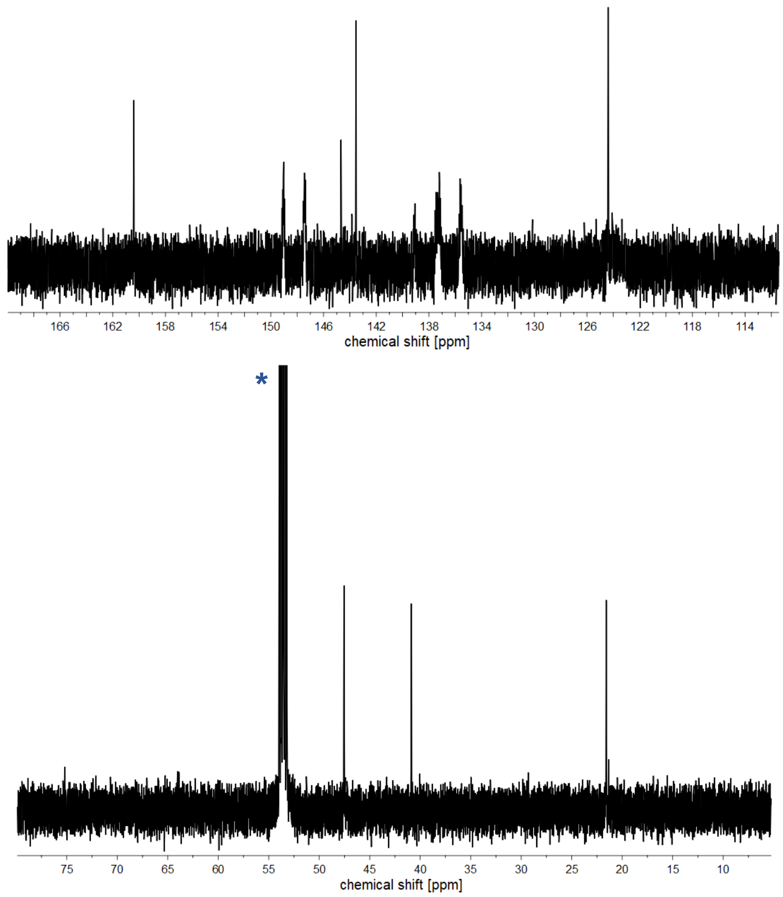

Figure S28. ^{13}C NMR (151 MHz, CD_2Cl_2) spectrum of **4**[B(C_6F_5)$_4$]$_4$; top: region of the aromatic carbons; bottom: region of the carbon signals of hpp. The peak marked by an asterisk denotes the solvent residual signal of CD_2Cl_2.

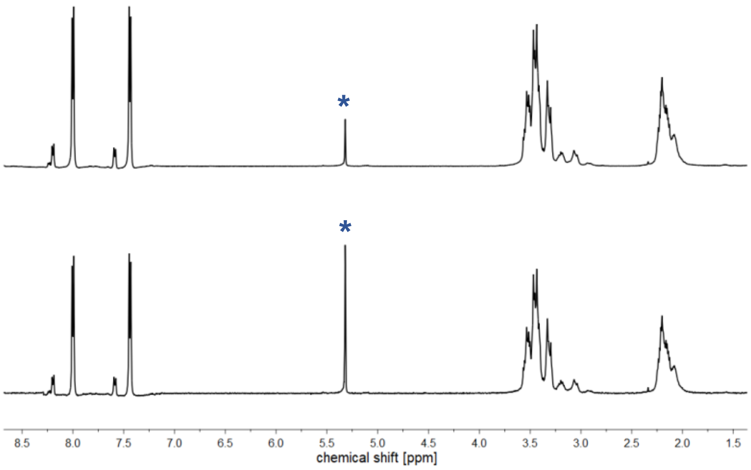

Figure S29. $^{1}H\{^{11}B\}$ NMR (400 MHz, CD_2Cl_2, top) and ^{1}H NMR (400 MHz, CD_2Cl_2, bottom) spectra of of **4**[B(C_6F_5)$_4$]$_4$ The peaks marked by an asterisk denote the solvent residual signal of CD_2Cl_2.

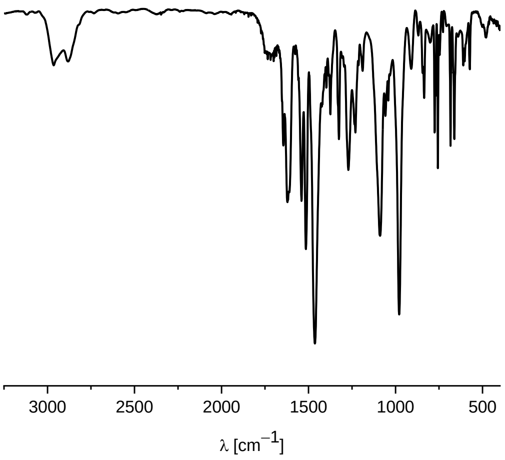

Figure S30. IR spectrum of **4**[B(C_6F_5)$_4$]$_4$ as a KBr disk.

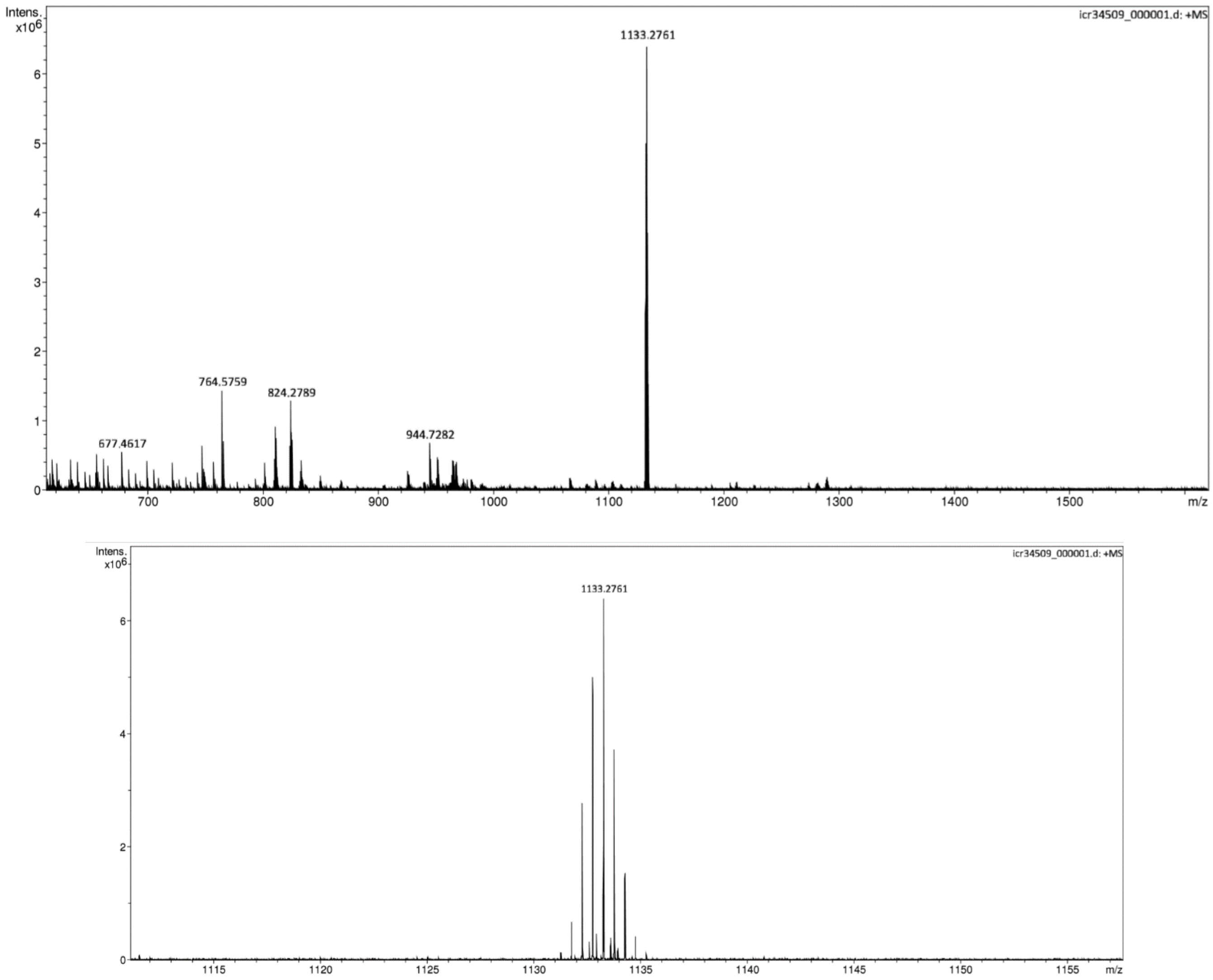

Figure S31. HR-ESI$^+$ mass spectrum of **4**[B(C_6F_5)$_4$]$_4$ recorded in a CH_2Cl_2 solution.

Analytical data for **5**(OTf)$_4$

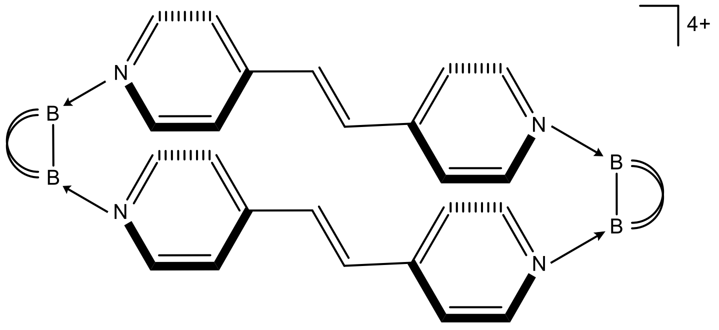

5 4 OTf$^-$

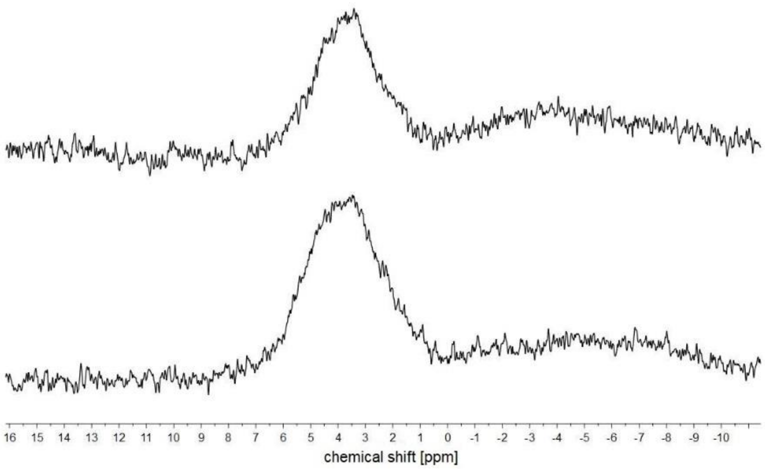

Figure S32. ^{11}B NMR (128 MHz, CD_3CN, top) and ^{11}B{^{1}H} NMR (128 MHz, CD_3CN, bottom) spectra of **5**(OTf)$_4$.

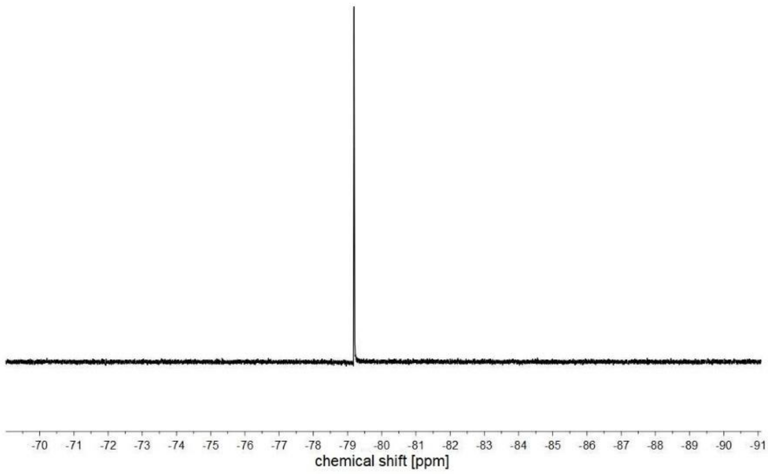

Figure S33. ^{19}F NMR (376 MHz, CD_3CN) spectrum of **5**(OTf)$_4$.

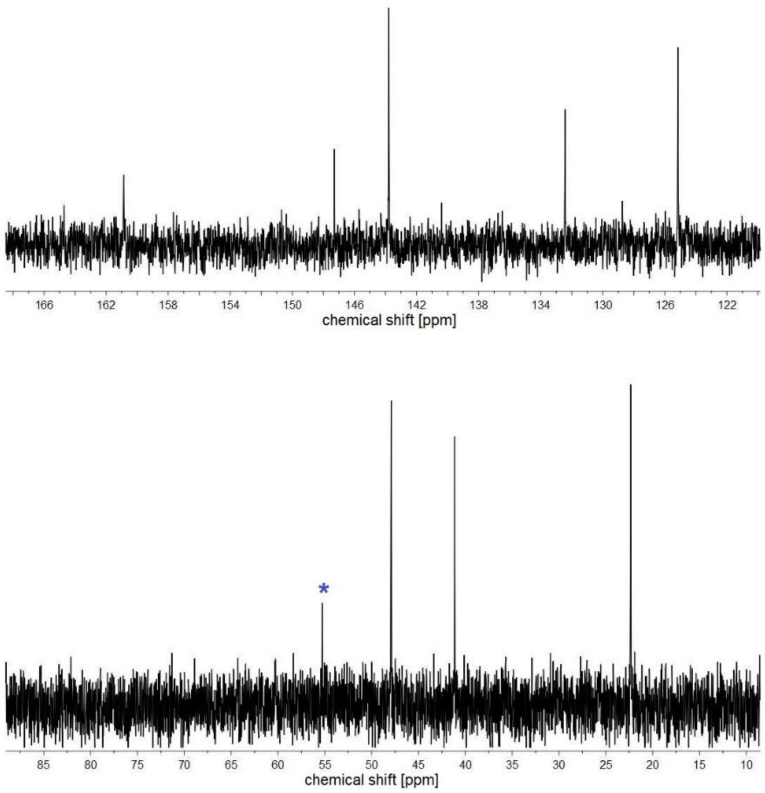

Figure S34. ^{13}C NMR (101 MHz, CD_3CN) spectrum of **5**(OTf)$_4$; top: region of the aromatic carbons; bottom: region of the carbon signals of hpp. The peak marked by an asterisk denotes the solvent residual signal of cocrystallized CH_2Cl_2.

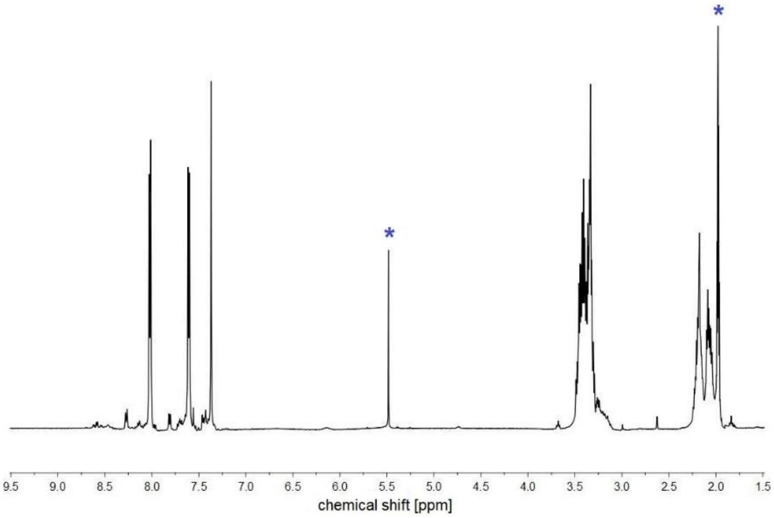

Figure S35. ^{1}H{^{11}B} NMR (400 MHz, CD_3CN) spectrum of **5**(OTf)$_4$. The peaks marked by an asterisk denote the solvent residual signals of CH_3CN and CH_2Cl_2.

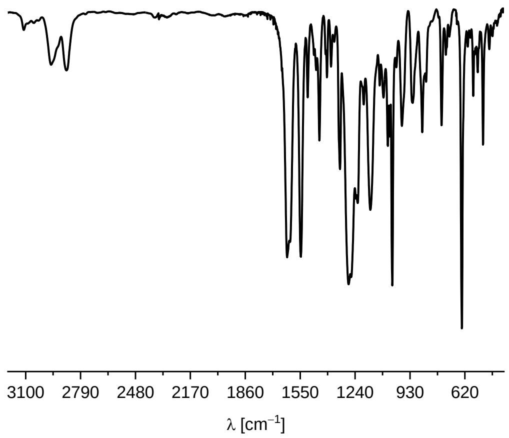

Figure S36. IR spectrum of **5**(OTf)$_4$ as a KBr disk.

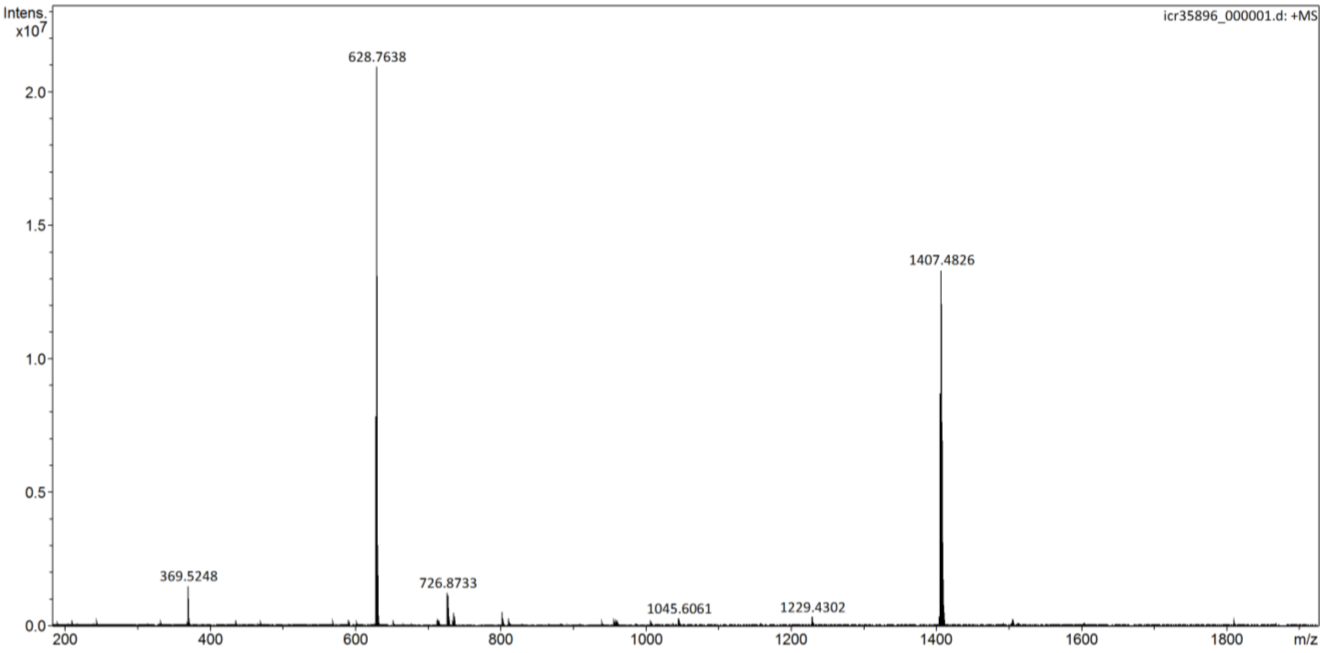

Figure S37. HR-ESI$^+$ mass spectrum of **5**(OTf)$_4$ recorded in a CH_2Cl_2/CH_3OH solution.

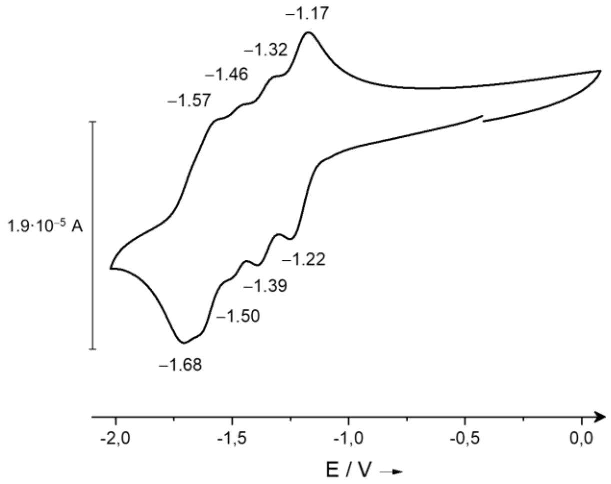

Figure S38. The cyclovoltamogramm of **5**(OTf)$_4$. A glassy carbon working electrode, a platinum counter electrode and an Ag/AgCl reference electrode were used to characterize a solution in CH_3CN at 298 K with 0.1 M $TBAPF_6$ serving as the electrolyte. A scan rate of 20 mV s^{-1} was used for this analysis and the obtained values were referenced to the ferrocenium/ferrocene (Fc^+/Fc) redox pair.

Analytical data for **6**$(OTf)_2$

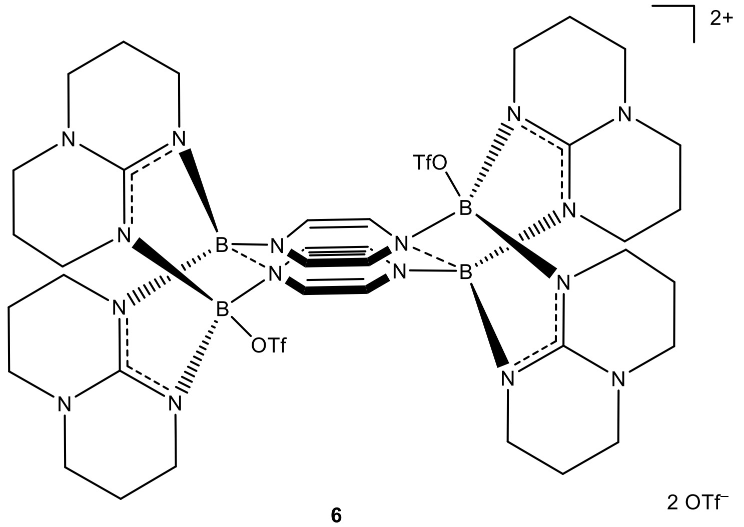

6

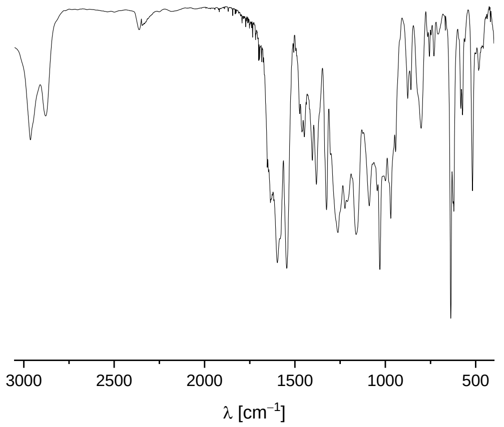

Figure S39. IR spectrum of **6**$(OTf)_2$ as a KBr disk.

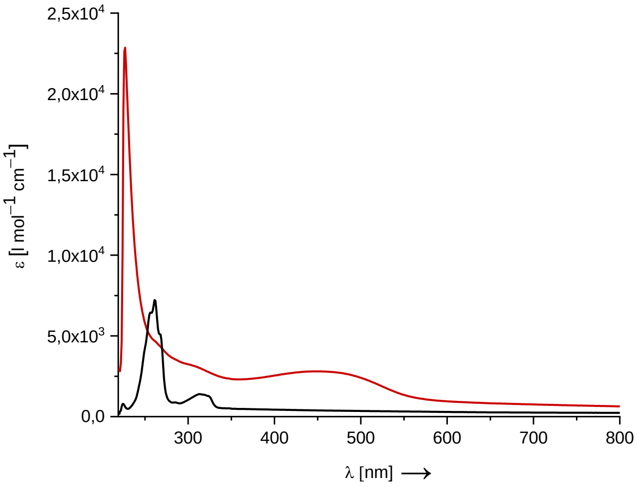

Figure S40. UV/Vis spectrum of **6**$(OTf)_2$ in CH_2Cl_2 at room temperature (red); c = 4.0 · 10^{-5} mol l^{-1}; λ_{max}/nm (ε in l mol^{-1} cm^{-1}) = 449 (2.8 · 10^3), 301 (3.2 · 10^3), 227 (2.3 · 10^4) and the spectrum of the starting material 1,4-diazine (black).

Analytical data for **7**(OTf)$_2$

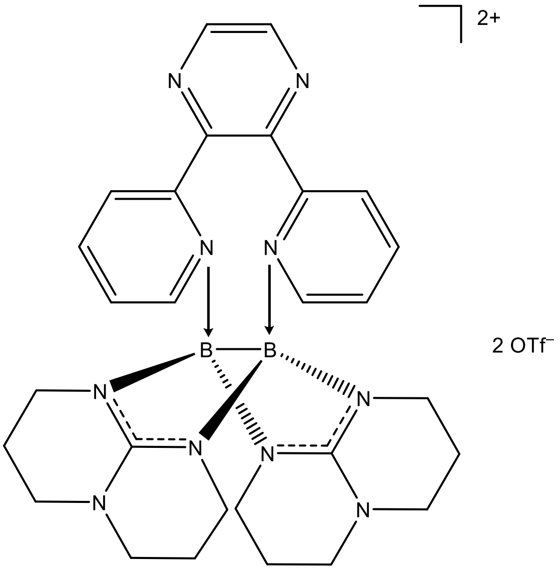

7

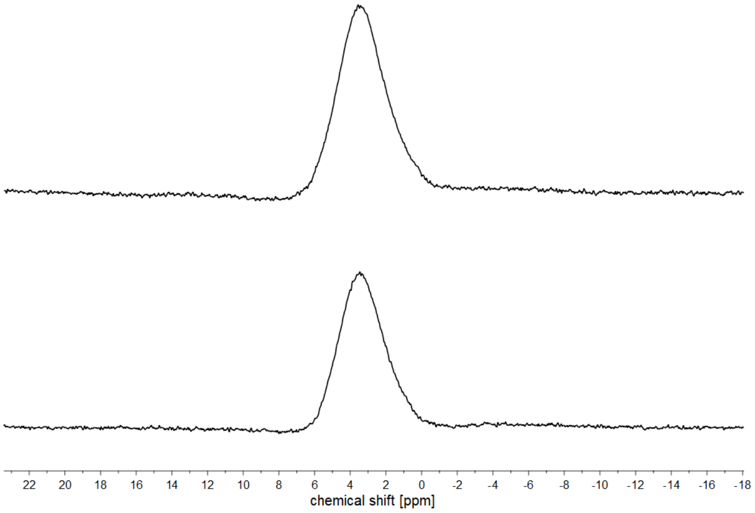

Figure S41. ^{11}B NMR (128 MHz, CD_2Cl_2, top) and ^{11}B{^{1}H} NMR (128 MHz, CD_2Cl_2, bottom) spectra of **7**(OTf)$_2$.

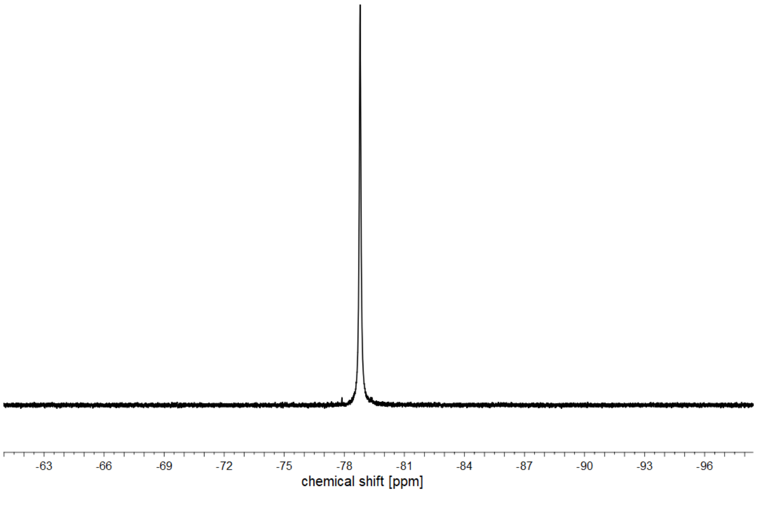

Figure S42. ^{19}F NMR (376 MHz, CD_2Cl_2) spectrum of **7**(OTf)$_2$.

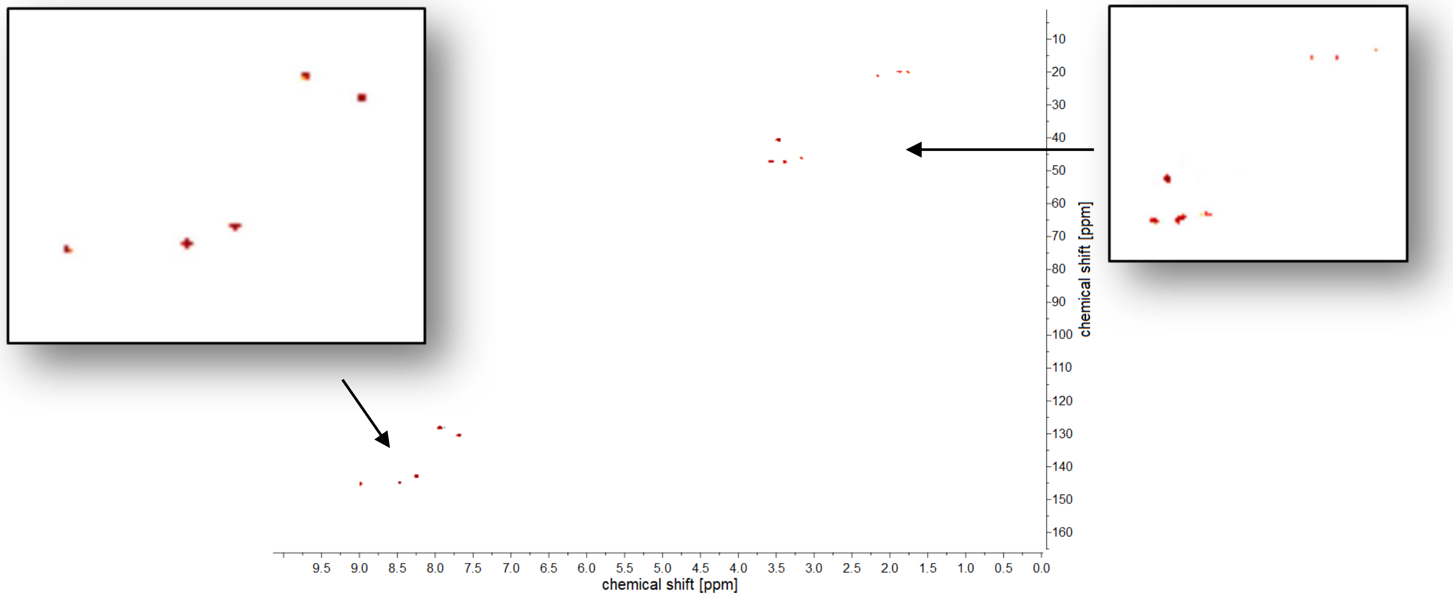

Figure S43. ^{1}J CH-Korr (HSQC) NMR (400 MHz, CD_2Cl_2) spectrum of **7**$(OTf)_2$.

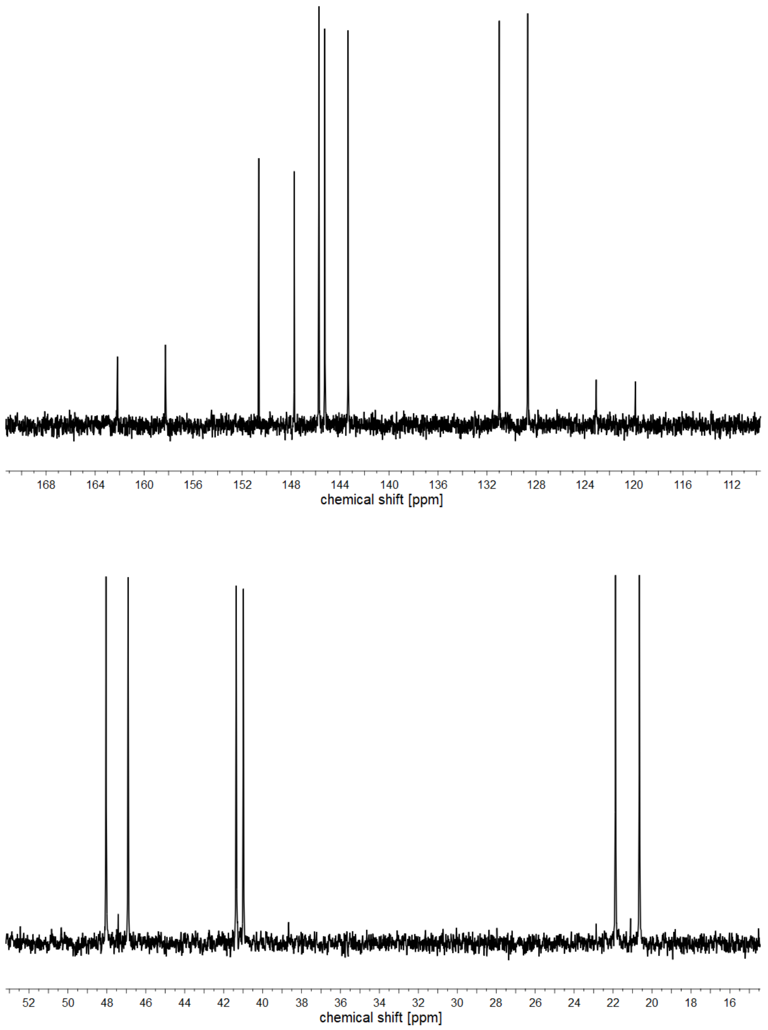

Figure S44. ^{13}C NMR (151 MHz, CD_2Cl_2) spectrum of **7**$(OTf)_2$; top: region of the aromatic carbons; bottom: region of the carbon signals of hpp.

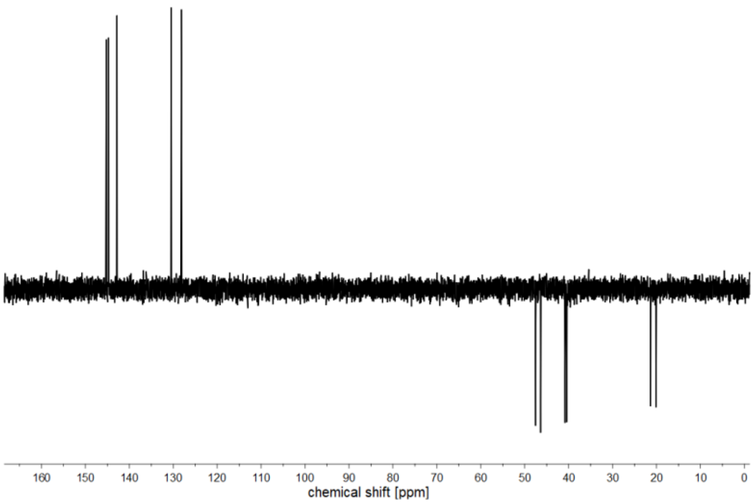

Figure S45. $^{31}C\{^{1}H\}$ DEPT135 NMR (101 MHz, CD_2Cl_2) spectrum of **7**$(OTf)_2$.

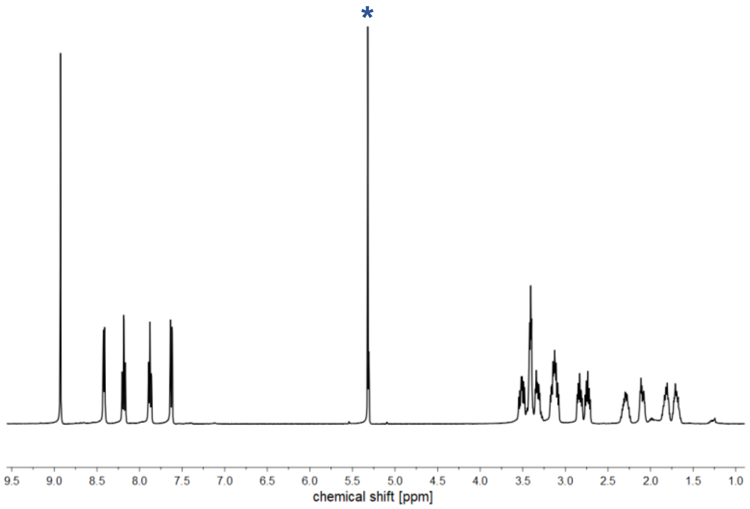

Figure S46. ^{1}H NMR (400 MHz, CD_2Cl_2) spectrum of **7**$(OTf)_2$. The peak marked by an asterisk denotes the solvent residual signal of CD_2Cl_2.

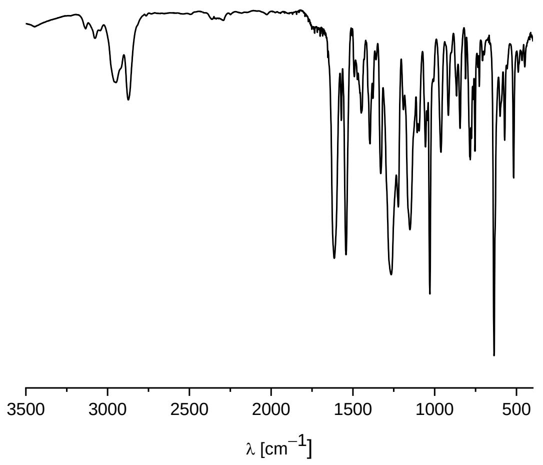

Figure S47. IR spectrum of **7**$(OTf)_2$ as a KBr disk.

Details of the quantum-chemical calculations

DFT calculations were performed with the TURBOMOLE V. 7.2[15] or 7.3.1[16–24] and ORCA V. 4.1.1[25] suite of programs. Geometry optimizations were performed at the RI-DFT[26] level of theory with a DFT-D3 dispersion correction[27] using the BP86[28] or B3LYP[29] functional in combination with the def2-SVP or def2-TZVPP[30,31] basis set. The vibrational frequencies of all optimized structures were calculated to verify if the optimized geometry represents a global energy minimum. All calculated structures were visualized with the GaussView 5.0.9 software.[32]

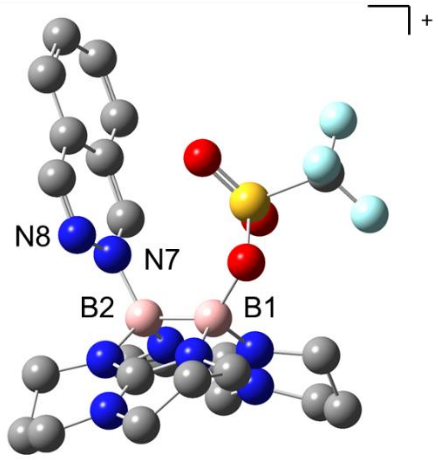

Figure S48. Calculated energy minimum structure (BP86/def2-SVP) of the monocationic monophthalazine-diborane adduct (hydrogen atoms omitted for clarity). Selected bond distances in Å: B1-B2 1.7601, B2-N7 1.5695, N7-N8 1.3638.

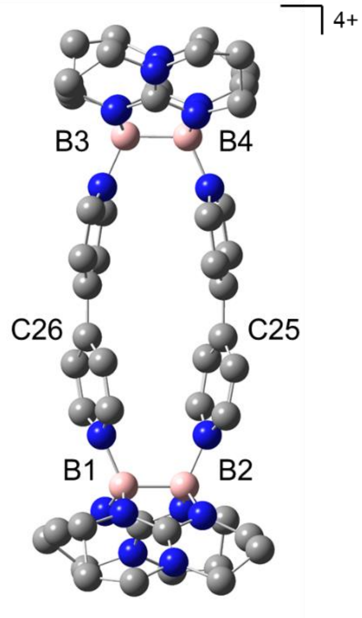

Figure S49. Calculated energy minimum structure (B3LYP-D3/def2-TZVPP) of the tetracationic 4,4'-bipyridyl cyclophane **4**$^{4+}$ (hydrogen atoms omitted for clarity). Selected bond distances in Å: B1-B2 1,7628, B3-B4 1.7625, C25-C26 3.8657.

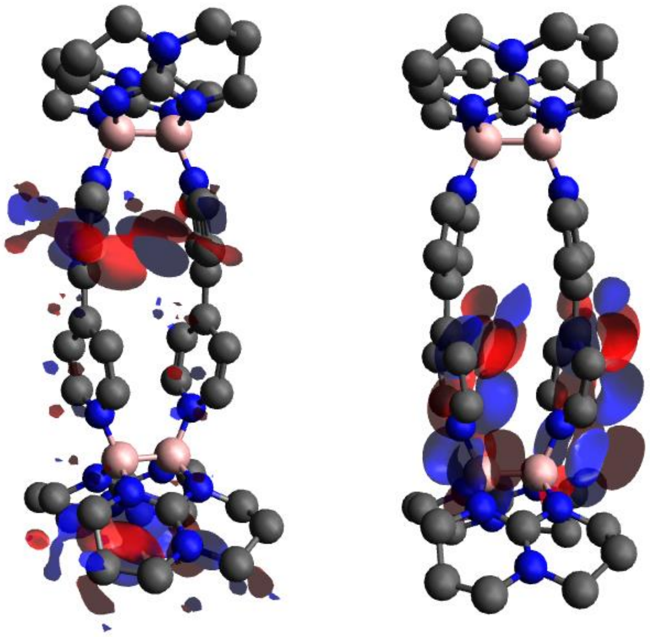

Figure S50. Illustration of the isodensity surfaces for the HOMO (left) and LUMO (right) of dicationic **4** obtained from the B3LYP-D3/def2-TZVPP calculation (isovalues of 0.025 au; atom colorcode: boron pink, nitrogen blue and carbon grey). Hydrogen atoms are omitted for clarity

.

References

[1] J. Horn, A. Widera, S. Litters, E. Kaifer, H.-J. Himmel, *Dalton Trans.* **2018**, *47*, 2009–2017.

[2] K. Kabsch, in: M. G. Rossmann, E. Arnold (eds.) *"International Tables for Crystallography" Vol. F*, Ch. 11.3, Kluwer Academic Publishers, Dordrecht, The Netherlands, **2001**.

[3] *CrysAlisPro*, Agilent Technologies UK Ltd., Oxford, UK **2011**–**2014** and Rigaku Oxford Diffraction, Rigaku Polska Sp.z o.o., Wrocław, Poland **2015**–**2019**.

[4] R. H. Blessing, *Acta Cryst.* **1995**, *A51*, 33.

[5] *SCALE3 ABSPACK, CrysAlisPro*, Agilent Technologies UK Ltd., Oxford, UK **2011**–**2014** and Rigaku Oxford Diffraction, Rigaku Polska Sp.z o.o., Wrocław, Poland **2015**–**2019**.

[6] W. R. Busing, H. A. Levy, *Acta Cryst.* **1957**, *10*, 180.

[7] C. Giacovazzo *"Phasing in Crystallography"*, IUCr and Oxford Science Publications, Oxford, UK, **2013**.

[8] (a) M. C. Burla, R. Caliandro, B. Carrozzini, G. L. Cascarano, C. Cuocci, C. Giacovazzo, M. Mallamo, A. Mazzone, G. Polidori, D. Siliqi *SIR2019*, CNR IC, Bari, Italy, **2019**; (b) M. C. Burla, R. Caliandro, B. Carrozzini, G. L. Cascarano, C. Cuocci, C. Giacovazzo, M. Mallamo, A. Mazzone, G. Polidori, *J. Appl. Cryst.* **2015**, *48*, 306.

[9] (a) L. Palatinus, *SUPERFLIP*, EPF Lausanne, Switzerland and Fyzikální ústav AV ČR, v. v. i., Prague, Czech Republic, **2007**–**2014**; (b) L. Palatinus, G. Chapuis, *J. Appl. Cryst.* **2007**, 40, 786.

[10] (a) G. M. Sheldrick, *SHELXL-20xx*, University of Göttingen and Bruker AXS GmbH, Karlsruhe, Germany **2012**–**2018**; (b) W. Robinson, G. M. Sheldrick in: N. W. Isaaks, M. R. Taylor (eds.) *„Crystallographic Computing 4"*, Ch. 22, IUCr and Oxford University Press, Oxford, UK, **1988**; (c) G. M. Sheldrick, *Acta Cryst.* **2008**, *A64*, 112; (d) G. M. Sheldrick, *Acta Cryst.* **2015**, *C71*, 3.

[11] (a) J. S. Rollett in: F. R. Ahmed, S. R. Hall, C. P. Huber (eds.) *„Crystallographic Computing"* p. 167, Munksgaard, Copenhagen, Denmark, **1970**; (b) D. Watkin in: N. W. Isaaks, M. R. Taylor (eds.) *„Crystallographic Computing 4"*, Ch. 8, IUCr and Oxford University Press, Oxford, UK, **1988**; (c) P. Müller, R. Herbst-Irmer, A. L. Spek, T. R. Schneider, M. R. Sawaya in: P. Müller (ed.) *"Crystal Structure Refinement"*, Ch. 5, Oxford University Press, Oxford, UK, **2006**; (d) D. Watkin, *J. Appl. Cryst.* **2008**, *41*, 491.

[12] A. Thorn, B. Dittrich, G. M. Sheldrick, *Acta Cryst.* **2012**, *A68*, 448.

[13] (a) P. v. d. Sluis, A. L. Spek, *Acta Cryst.* **1990**, *A46*, 194; (b) A. L. Spek, *Acta Cryst.* **2015**, *C71*, 9.

[14] (a) A. L. Spek, *PLATON*, Utrecht University, The Netherlands; (b) A. L. Spek, *J. Appl. Cryst*. **2003**, *36*, 7.

[15] Turbomole V. 7.2, Karlsruhe, Germany, **2017**. R. Ahlrichs, M. Baer, M. Haeser, H. Horn, C. Koelmel, *Chem. Phys. Lett.* **1989**, *162*, 165.

[16] Turbomole V. 7.3.1, Karlsruhe, Germany, **2019**. R. Ahlrichs, M. Baer, M. Haeser, H. Horn, C. Koelmel, *Chem. Phys. Lett.* **1989**, *162*, 165.

[17] G. Schmitz, C. Hättig, D. Tew, *Phys. Chem. Chem. Phys*. **2014**, *16*, 22167–22178.

[18] a) E. Caldeweyher, C. Bannwarth, S. Grimme, *J. Chem. Phys.* **2017**, *147*, 034112; b) E. Caldeweyher, S. Ehlert, A. Hansen, H. Neugebauer, S. Grimme, *J. Chem. Phys.* **2018**, in progress; c) C. Bannwarth, S. Ehlert, S. Grimme, *J. Chem. Theory Comput*. **2018**, in progress.

[19] K. Reiter, F. Mack, F. Weigend, J. Chem. Theory Comput., **2018**, *14*(1), 191–197.

[20] S. M. Parker, D. Rappoport, F. Furche, *J. Chem. Theory Comput.* **2018***, 14*, 807–819.

[21] Y. J. Franzke, N. Middendorf, F. Weigend, *J. Chem. Phys*. **2018**, *148*, 104110.

[22] E. Tapavicza, F. Furche, D. Sundholm, *J. Chem. Theory Comput*. **2016**, *12*(10), 5058–5066.

[23] S. K. Khani, A. M. Khah, C. Haettig, *Phys. Chem. Chem. Phys*. **2018**, *20*, 16354–16363.

[24] C. Holzer, W. Klopper, to be published.

[25] ORCA V. 4.1.1, Mühlheim a. d. Ruhr, Germany, **2019**.

[26] a) K. Eichkorn, O. Treutler, H. Oehm, M. Häser, R. Ahlrichs, *Chem. Phys. Lett.* **1995**, *240*, 283; b) K. Eichkorn, O. Treutler, H. Oehm, M. Häser, R. Ahlrichs, *Chem. Phys. Lett.* **1995**, *242*, 652; c) K. Eichkorn, F. Weigend, O. Treutler, R. Ahlrichs, *Theo. Chem. Acc.* **1997**, *97*, 119.

[27] S. Grimme, J. Anthony, S. Ehrlich, H. Krieg, *J. Chem. Phys*. **2010**, *132*, 154104.

[28] a) A. D. Becke, *Phys. Rev. A: At. Mol. Opt. Phys.* **1988**, *38*, 3098–3100; b) J. P. Perdew, *Phys. Rev. B: Condens. Matter* **1986**, *33*, 8822–8824.

[29] C. Lee, W. Yang, R. G. Parr, *Phys. Rev. B* **1988**, *37*, 785–789.

[30] A. Schäfer, H. Horn, R. Ahlrichs, *J. Chem. Phys.* **1992**, *97*, 2571–2577.

[31] F. Weigend, M. Häser, H. Patzelt, R. Ahlrichs, *Chem. Phys. Lett.* **1998**, *294*, 143–152.

[32] GaussView 5.0.9, R. D. Dennington II, T. A. Keith, J. M. Millam, **2000**–**2008**.
